# Self-Assembled Organic Materials for Photovoltaic Application

**DOI:** 10.3390/polym9030112

**Published:** 2017-03-21

**Authors:** Tanwistha Ghosh, Jayanthy S. Panicker, Vijayakumar C. Nair

**Affiliations:** 1 Photosciences and Photonics Section, Council of Scientific and Industrial Research—National Institute for Interdisciplinary Science and Technology (CSIR-NIIST), Trivandrum 695019, India; tanwistha.myrtus@gmail.com (T.G.); jayanthykuttickal@gmail.com (J.S.P.); 2 Academy of Scientific and Innovative Research (AcSIR), New Delhi 110001, India

**Keywords:** self-assembly, organic photovoltaic cells, bulk-heterojunction, donor-acceptor systems, oligomers, polymers

## Abstract

Organic photovoltaic cells based on bulk-heterojunction architecture have been a topic of intense research for the past two decades. Recent reports on power conversion efficiency surpassing 10% suggest these devices are a viable low-cost choice for a range of applications where conventional silicon solar cells are not suitable. Further improvements in efficiency could be achieved with the enhanced interaction between the donor and acceptor components. Effective utilization of supramolecular interactions to tailor and manipulate the communication between the components in the blend is a good strategy towards this end. Literature reports suggest that the long-term stability of organic solar cells, a major hurdle for commercial applications, can also be partially addressed by generating stable supramolecular nanostructures. In this review, we have made an attempt to summarize advances in small molecule, oligomer and polymer based systems, wherein supramolecular interactions such as hydrogen-bonding, pi-pi stacking, and dipole-dipole are explored for realizing stable and efficient bulk-heterojunction solar cells.

## 1. Introduction

Solar radiation is a prime source of energy and its abundant availability on Earth’s surface makes it a perfect candidate for clean and renewable sources of electricity [1,2,3]. Development in the field of solar energy harvesting will help to reduce the world’s dependence on exhaustible, expensive and polluting sources of energy, thereby help countries tackle their manifold energy challenges. Scientists are therefore putting enormous efforts in the field of photovoltaic technology as it can serve as the most high-potential prospect for harnessing solar energy. Silicon solar cells, the first generation solar technology, can convert ~25% of the energy in sunlight to electricity and the second generation cells composed of amorphous silicon, cadmium telluride (CdTe) and copper indium gallium selenide (CIGS) shows typical performance in the range of ~22% [4,5,6]. Though both these photovoltaic technologies have good performance and stability, they need highly expensive clean-room technology and require a lot of energy in production [7]. To address these problems, scientists have done intense research in the field of third generation photovoltaics, particularly organic solar cells (OSC) as it has the potential to serve as an alternative to silicon-based cells in terms of cost effectiveness, light weightiness, roll-to-roll fabrication, mechanical flexibility in addition to the use of low-cost solution processing techniques for large area fabrication [8,9,10,11,12,13]. Most of the current research is focusing toward the development of novel materials with improved efficiencies through molecular engineering [14,15,16,17,18]. Recent reports suggest that OSCs have entered the phase of commercialization, although improvements in efficiency and stability are still desirable [19,20,21].

**Figure 1:**
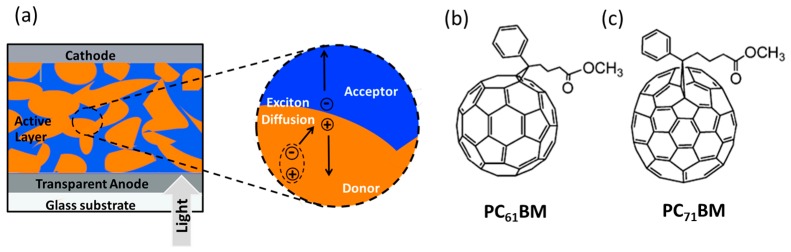
(**a**) Simplified schematic representation describing the main processes in a bulk-heterojunction solar cell device. The light absorption by the active layer (predominantly donor) generates excitons, which diffuses towards the donor-acceptor interface where they separate into free charge carriers (holes and electrons). Holes move towards anode and electrons move to the cathode. Chemical structures of fullerene-based acceptors (**b**) PC_61_BM and (**c**) PC_71_BM.

Organic solar cells consist of organic semiconductors such as small molecules, oligomers and polymers as active photoabsorption layer. The incorporation of both donor and acceptor type semiconductors in the photoabsorption layer is the most common strategy, as it can split excitons more efficiently than single layer photovoltaic cells. This idea started with the phenomenal work by Tang in 1986, where he demonstrated efficient charge separation at the donor-acceptor interface (bilayer architecture), yielding a power conversion efficiency (PCE) of 1% [22]. It is a well-known fact that the charge carrier recombination has an adverse effect on the efficiency of OSCs. For efficient photoabsorption in a bilayer solar cell, the layers are required to have a thickness of at least 100 nm but the excitons may not be able to travel more than 10–20 nm before recombination [23]. As a result, most of the excitons generated in bilayer devices do not reach the interface and contribute to the charge carrier generation. Therefore, the PCEs of the bilayer solar cells usually lie in the range of 1.0%–1.5% [24,25] and approach a maximum of 2%–5% in exceptional cases [26,27,28,29,30]. Intermixing of donor and acceptor was proposed as a plausible solution to solve this problem as the thickness of the light absorbing material can be 100 nm or above but the creation of nanodomains of the order of 10–20 nm facilitates efficient dissociation of the generated excitons to charge carriers (holes and electrons). These charges can travel to the respective electrodes (holes to the anode and electrons to the cathode) through the interpenetrating donor/acceptor network with minimum recombination. This active layer architecture is known as bulk-heterojunction (BHJ), which is schematically shown in Figure 1a. The first prototype of this kind was realized and experimentally verified by Heeger and co-workers in 1992 [31]. The donors used in BHJ solar cells are normally pi-conjugated small molecules, oligomers or polymers and commonly used acceptors are soluble derivatives of fullerene such as phenyl-C61-butyric acid methyl ester (PC_61_BM; Figure 1b) and phenyl-C71-butyric acid methyl ester (PC_71_BM; Figure 1c). The major requirement of the donor is to have an optical band gap in the visible range of the solar spectrum. Light falling on the active layer is therefore efficiently absorbed by the donor and leads to the generation of highly energetic excitons (ca. 100 MeV). For the efficient diffusion and dissociation of excitons and the transport of charge carriers, the active layer should have a bicontinuous interpenetrating network of donors and acceptors with a minimum number of defect sites. Rational use of supramolecular interactions is considered as an ideal strategy towards this direction.

### 1.1. Supramolecular Interactions and Self-Assembly

Supramolecular chemistry, which deals with non-covalent interactions, is one of the frontier fields of chemistry in the 21st century. This area, initially developed by Jean-Marie Lehn, Donald Cram and Charles Pedersen, is defined as the “chemistry of molecular assemblies and of the intermolecular interactions” [32]. Contrary to covalent interactions, the sharing of electrons does not take place in supramolecular, but rather involves an electrostatic interaction between molecules or within a molecule. The process by which molecules adopt a defined or ordered arrangement using various non-covalent interactions without the influence from an outside source is called self-assembly. In this process, small components (individual building blocks) join together spontaneously and form large and complex supramolecular assemblies. Major non-covalent forces involved are hydrogen bonding, ion-ion, ion-dipole, dipole-dipole, pi-pi stacking, cation-pi, anion-pi, van der Waals and hydrophobic interactions [33].

#### 1.1.1. Hydrogen Bonding

Hydrogen bonding (H-bonding), a weak intermolecular interaction (4–60 kJ/mol), was first noted by Nernst in 1892, is an attractive force between the hydrogen attached to an electronegative atom of one molecule and an electron lone pair on another electronegative atom of a different molecule [34]. Such an interaction was little known at that time but later in 1935 its actual mechanism was extricated and the term “hydrogen bond” was proposed by two scientists, Bernal and Huggins. The best use of H-bonding in nature includes the folding of the protein and double helical structure of DNA. Since then, H-bonding interactions have continued to fascinate chemists, biologists, material scientists and theoreticians across the globe.

Hydrogen-bonding is directional in nature and hence the hydrogen-bond donor, the hydrogen atom and the hydrogen-bond acceptor should lie in the same line for obtaining strong bonds. However, the stability of single bonded hydrogen bonding sites limits their direct usage in the non-covalent synthesis of well-defined assemblies. Therefore, different strategies have been adopted to increase the strength, which include the use multiple H-bonding motifs. The stability of multiple H-bonded motifs was studied by Jorgensen and co-workers. The “Jorgensen model” states that the total hydrogen bond should be more stable when all the protons of the H-bond donor-acceptor pairs are arranged in the same direction. On the other hand, the H-bond should be less stable when the protons are arranged alternatively [35,36].

#### 1.1.2. Ion-Ion Interactions

As the name suggests, the ion-ion interactions occur between charged atoms or molecules. The strength of ionic bonding is comparable to that of covalent bonds with bond energy approximately 100–350 kJ/mol. These bonds are harder to break and therefore it is the strongest bond among all supramolecular interactions. However, this interaction can be easily broken with the addition of water or any other polar solvent. The best example of a supramolecular compound with ion-ion interaction is sodium chloride (NaCl). The energy required to break the bond in NaCl is 188 kcal/mol as a result of which it has a boiling point of 1413 °C.

#### 1.1.3. Ion-Dipole Interactions

Here, the bonding is between an ion and a molecule with a permanent or induced dipole moment, and the energy typically lies between 50 and 200 kJ/mol. This kind of bonding is observed both in solids as well as in solutions. The interaction between an electrophilic interior of a crown ether and a cation is a typical example of this kind. Co-ordinate bonds are included in this category as these interactions are between nonpolarisable metal cations and hard bases. Two main types of ion-dipole interactions are orientation interaction (ion—permanent dipole) and induction interaction (ion—induced dipole).

#### 1.1.4. Dipole-Dipole Interactions

The attraction between the positive and negative ends of two neighboring molecules is termed as dipole-dipole interaction. It mostly involves molecules having a permanent dipole moment. These electrostatic interactions induce alignment of the molecules to increase the attraction between assemblies and are considered as a type of van der Waals forces. The dipoles are normally associated with electronegative atoms, such as oxygen, nitrogen, sulfur, and fluorine. The strength of the dipole-dipole interaction depends on (a) magnitude of the interacting dipoles; (b) distance between the centers of the two interacting dipoles; (c) the angle of orientation of two parallel dipoles and (d) the overlap integral between energy states which indirectly satisfies the conservation of momentum and energy. The Debye force or the permanent-induced dipoles interaction is another type interaction comes under the same category, which involves an interaction between molecules with a permanent dipole to another molecule with no permanent dipole. London dispersion forces, the weakest type of non-covalent interaction, are most commonly known as “induced-induced dipoles interaction” and present between all molecules.

#### 1.1.5. Pi-Pi Stacking Interactions

The interaction that is frequently observed for stabilizing non-covalent interactions between parallel aromatic rings is “pi-pi stacking”. The concept of pi-pi stacking of organic semiconducting materials was first established by Sanders and Hunter in the early 1990s [37]. They have reported the formation of a partial negative quadruple moment above the aromatic planes and a partial positive charge around the periphery due to the pi-electron cloud density on the aromatic rings. They have used a simple mathematical model to qualitatively determine the effect of substituents. According to their model, parallel displaced and sandwich conformations are favored for aromatic rings containing electron-withdrawing groups as they reduce the negative quadruple moment. On the other hand, a T-shaped configuration is preferred for aromatic molecules with electron donating groups. Such an interaction helps to reduce the steric hindrance caused by non-conjugated side chains.

#### 1.1.6. Cation-Pi Interactions

Ferrocene and Zeise’s salt are typical examples of cation-pi interaction, where transition metal cations such as Fe^2+^ and Pt^2+^ form complexes with olefinic and aromatic hydrocarbon. In general, it is a kind of non-covalent bonding between a cation and a quadrupole (pi-system), where the negatively charged region of aromatic ring quadrupole interact favorably with positively charged species (cation). The interaction of alkaline and alkaline earth metal cations with carbon-carbon double bond is one of the most studied cation-pi interaction. Dougherty and co-workers reported an electrostatic model based on differences in electrostatic attraction for describing the trends in binding energy. It was understood that interaction energies of cation-pi pairs correlate well with electrostatic potential above the pi-face of arenes.

#### 1.1.7. Anion-Pi Interactions

As the name suggest, it is an attraction between an anion and pi-electron density. Intuitively, it should be repulsive. However, a small charge difference occurs between a neutral aromatic ring and an anion, and in principle, an electrostatic attraction exists between them. This interaction is a reason for the self-assembly reactions of pi-acidic aromatic rings with Ag(I) complexes. In 2004, the first anion-pi interaction was reported in the crystal structure of carousel copper(II)-triazine complex [38]. Though this kind of interaction is depicted in the solid state, there is evidence that the interaction is present in solution also.

#### 1.1.8. Van Der Waals Forces

Van der Waals interaction, named after Dutch physicist, Johannes Diderik van der Waals, is a very weak, short-range electrostatic attraction independent of temperature arises from the polarization of electron cloud by the propinquity of an adjacent nucleus. Due to its non-directional characteristic, the scope of designing specific hosts utilizing van der Waals interaction is limited for the selective complexation of a particular guest. The geckos stick on to the wall is an example of van der Waals forces from nature.

#### 1.1.9. Hydrophobic Interactions

Hydrophobic interactions mainly occur between non-polar molecules or more specifically hydrocarbons in an aqueous environment. Water is comprised of a three-dimensional network of hydrogen bonds (~5 kcal/mol) which are dynamic in nature. When non-polar molecules come in the vicinity of hydrophobic surfaces, water molecules will drive them to aggregate. This process can significantly decrease the energetically unfavorable interface. Such hydrophobic interactions are omnipresent in biological systems and are responsible for key natural processes such as protein folding, membrane formation, etc.

It is a well-known fact that the morphology of active layers plays a crucial role in the power conversion efficiency of BHJ based organic solar cells [39,40]. It influences almost all aspects of the device like charge carrier generation, recombination, mobility and collection efficiency. Therefore, enabling a control over morphology will help in unlocking the vast potential of OSCs into viable technologies. Scientists have made attempts to incorporate the above mentioned supramolecular interactions into organic molecules for solar cell applications to achieve control on morphology via optimized nanodomains resulting in precise bicontinuous donor-acceptor networks and improved stability. The most commonly employed interactions in a solar cell so far are hydrogen bonding, pi-pi stacking, and dipole-dipole. In the following sections, we will discuss those works which have highlighted the self-assembly of donors and/or acceptors using non-covalent forces for solar cell applications. A number of reviews are available in literature discussing various aspects of supramolecular systems for solar cell application [41,42,43,44]. Most of the reviews highlight the design principles and strategies for morphology control using supramolecular interactions to get improved efficiency and stability. The present review provides a comprehensive summary of BHJ based solar cell devices consisting of self-assembled supramolecular systems (small molecules, oligomers and polymers). This review is structured around the various intermolecular forces and their effect on device performance. However, we will not be discussing papers on regular pi-conjugated systems used in bulk-heterojunction solar cells having obvious pi-pi stacking interactions. We have taken into account of those works where self-assembly is induced via special molecular design such as the introduction of hydrogen bonding groups, large pi-chromophores, dipolar moieties, etc.

## 2. Small Molecules and Oligomers

Small molecules and oligomers have earned intense scientific interest when compared to that of polymers for solar cell applications over the last few years due to their ease of preparation in high purity, no batch-to-batch variation and well-defined structure [45,46]. Recent reports suggest that the power conversion efficiency has reached above 10% by adopting BHJ architecture [47]. Important papers describing the self-assembly of the components of the active layer of a bulk-heterojunction organic solar cell through non-covalent interactions is discussed below.

**Figure 2:**
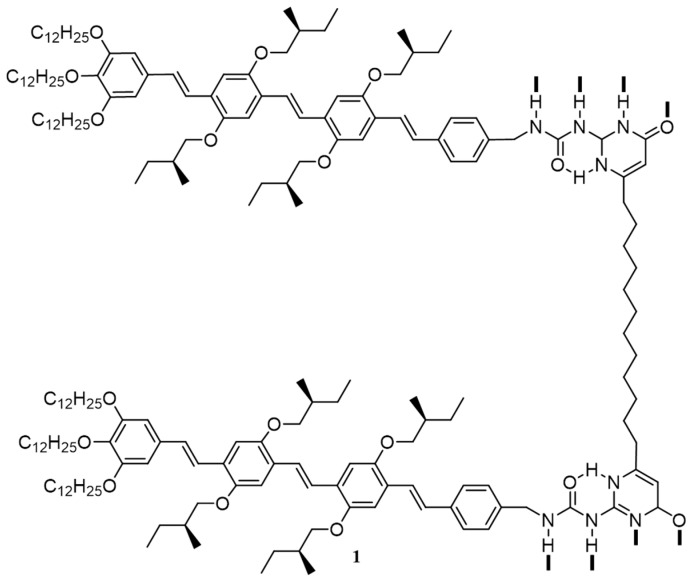
Chemical structure of the bifunctional ureido-pyrimidinone appended with oligo(*p*-phenylenevinylene) chromophores (**1**) synthesized by Janssen and Meijer et al. [48].

Early in 2001, Janssen, Meijer and co-workers for the first time synthesized and studied the electronic properties of a hydrogen-bonded pi-conjugated supramolecular system consisting of bifunctional ureido-pyrimidinone appended with oligo(*p*-phenylenevinylene) chromophores (**1**) as shown in Figure 2 [48]. The authors have also investigated the photovoltaic properties of the self-assembled molecules by mixing with PC_61_BM as an electron acceptor. Spin-coated blend films of **1**/PC_61_BM (1:2.8 by weight) showed complete quenching of the phenylenevinylene fluorescence. Fabricated devices with architecture, ITO/PEDOT:PSS/**1**:PC_61_BM/Al, yielded short circuit current of 0.32 mA·cm^−2^, open circuit voltage of 0.82 V and fill factor of 0.39. The values of short circuit current and open circuit voltage are favorable and comparable to previous reports on solar cells based on pi-conjugated systems in combination with fullerene. This work illustrated that supramolecular systems could act as promising materials for photovoltaic devices. This work has also opened up multiple opportunities to the usage of supramolecular architectures in optoelectronic devices.

**Figure 3:**
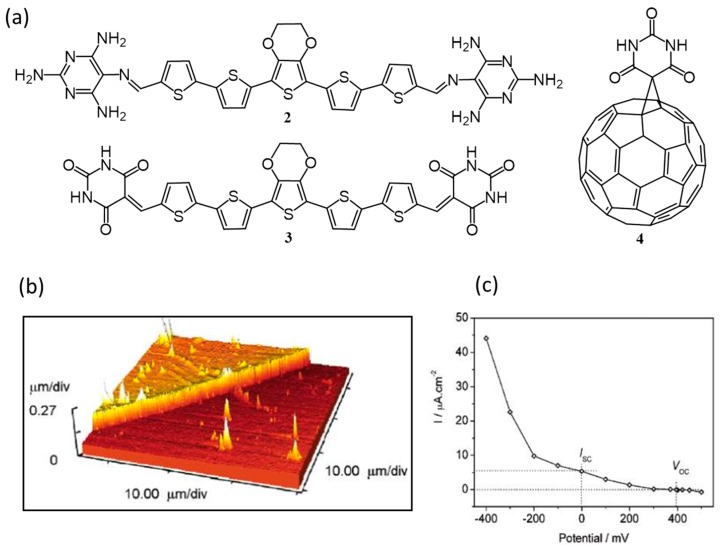
(**a**) Chemical structures of oligomers (**2** and **3**) and a complementary fullerene-substituted barbituric acid derivative (**4**) synthesized by Bassani et al. [49]. (**b**) Atomic force microscopy (AFM) image of the film of **2** prepared from DMSO. (**c**) *I*/*V* curve obtained from a drop-cast film of **2** on Au. Adapted with permission from Ref. [49] Copyright 2005 American Chemical Society.

The incorporation of non-self-complementary hydrogen bonding motifs present in melamine/barbituric acid moieties is found to be an excellent tool to control the geometries of electron donors and acceptors. In 2005, Bassani and co-workers had for the first time incorporated molecular recognition motifs based on these moieties into thiophene oligomers using Schiff base formation or the Knoevenagel condensation reactions [49]. They synthesized two oligomers with central ethylenedioxythiophene (EDOT) core and 2,4,5,6-tetraaminopyrimidine (**2**) or complementary barbituric acid (**3**) at the ends as shown in Figure 3a. With respect to the parent oligomer (pentathiophene), **2** and **3** showed better solubility and lower oxidation potential due to the presence of central EDOT unit. Photovoltaic devices were fabricated using **2** as a donor and a complementary fullerene-substituted barbituric acid derivative (**4**) as an acceptor. The performance of the devices was evaluated in terms of open-circuit voltage conversion efficiency, Ф(*V*_OC_), and short-circuit quantum conversion efficiency, Ф(*I*_SC_). Initially, the oligomer **2** alone was tested for a photovoltaic response and the obtained values of Ф(*V*_OC_) and Ф(*I*_SC_) were 0.14 and 0.05, respectively. Atomic force microscopy (AFM) image of the self-assembled film is shown in Figure 3b. The *I*/*V* curve of photocurrent generated vs. applied potential was plotted as given in Figure 3c. Devices were fabricated under identical conditions using **2** (1 equiv.) in combination with two equiv. of either pristine C_60_ or **4**. With reference to oligomer alone, two-fold increments in photocurrent were observed for the combination of **2**/C_60_ and five-fold increment for the combination of **2**/**4**. The better performance of **2**/**4** device could be attributed to the complementary H-bonding between the active components that granted extended organized domains, which helped in the charge separation and transport.

**Figure 4:**
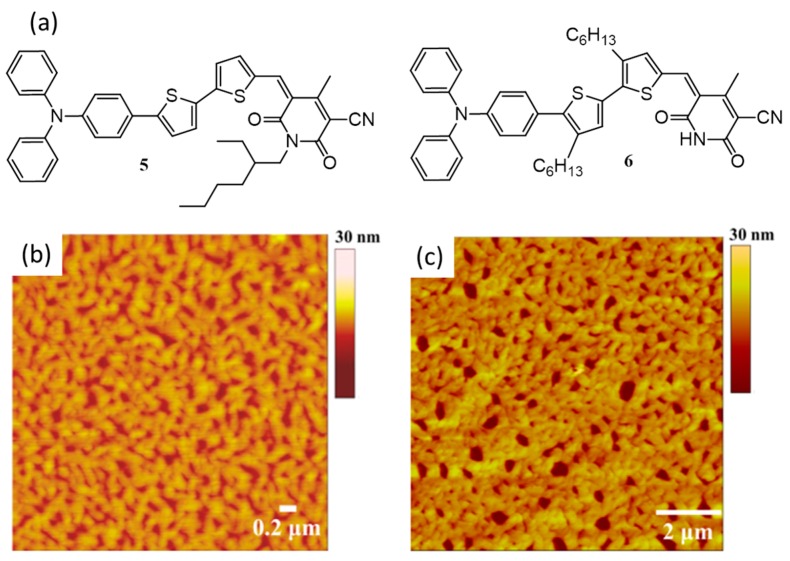
(**a**) Chemical structures of **5** and **6** synthesized by Kumar et al. [51]. AFM height image of observed nanostructures of films drop-cast from (**b**) 1 mg/mL and (**c**) 10 mg/mL chloroform solution of **6**. Adapted from Ref. [51] which is an Open Access article under the terms of the Creative Commons Attribution License (http://creativecommons.org/licenses/by/2.0).

Use of electron deficient, self-complementary hydrogen-bonding units incorporated as an acceptor in “push–pull” p-type small molecules were proposed as another method to enhance the efficiency of photovoltaic devices. Kumar and co-workers have reported a comparison study of two such donor–acceptor small molecules, **5** [50] and **6** (without and with self-complementary hydrogen-bonding units), as given in Figure 4a [51]. Both molecules possess cyanopyridone moiety as the acceptor motif, where the nitrogen is alkylated with a 2-ethylhexyl substituent in **5**, which masks the possibility of self-assembly while it is free or unmasked in the case of **6**. The formation of interconnected nanostructures of **6** was confirmed by AFM and ^1^H NMR analysis. AFM images of films prepared from 1 and 10 mg/mL chloroform solutions of **6** (Figure 4b,c) revealed the formation of interconnected fibrous nanostructures of ~85–110 nm in width. The UV-visible absorption spectra of a thin film of **6** show blue-shift of the absorption peak (30 nm) in comparison with the solution state indicated the formation of H-aggregates. Upon fabrication of BHJ cells using 1:1 mixture of donor and acceptor (PC_61_BM), **6** gave an efficiency of 2.40% at a concentration of 10 mg/mL, whereas, **5** yielded an efficiency of 2.25% at a higher concentration of 20 mg/mL. It was assumed that the hole percolation to the electrodes facilitated by the one-dimensional chains of **6** formed through hydrogen bonding created networks, resulting in an increase in PCE.

**Figure 5:**
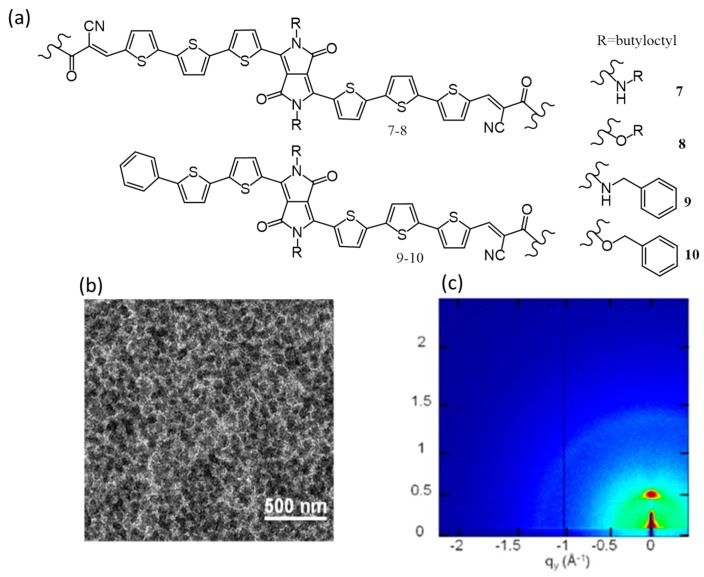
(**a**) Chemical structures of **7**–**10** synthesized by Stupp et al. [52]. (**b**) Transmission electron microscopy (TEM) image of **9**/PC_71_BM and (**c**) two-dimensional grazing incidence X-ray diffraction (2D-GIXRD) of **9** drop-cast from a mixture of CB and NB. Adapted with permission from Ref. [52] Copyright 2015 American Chemical Society.

An example to explain the ability of an amide group to provide hydrogen-bonding and a second driving force for self-assembly beyond pi-pi stacking was emphasized by the research group of Stupp in 2015 [52]. They have synthesized symmetric (**7**, **8**) and asymmetric (**9**, **10**) donor molecules (Figure 5a) to study the effect of H-bonding on the active layer morphology. Devices from **7** to **8** were fabricated from chlorobenzene (CB) using PC_71_BM as the acceptor with a blend ratio of 1:1. A poor efficiency value of 1% was observed for both molecules due to the formation of large aggregates (~100 nm), which limited the exciton diffusion to the interphase. Interestingly, devices made from asymmetric derivatives were better than the symmetric counterparts; PCE of **9** and **10** were 3.65 and 1.45%, respectively. In addition, the effect of solvent additives such as 1,8-diiodooctane (DIO) and nitrobenzene (NB) on device performance was also studied. The NB addition to **9** gave the best PCE of 4.57%. The performance enhancement upon addition of NB was due to the formation of more interconnected domains, as evident from the transmission electron microscopy (TEM) image (Figure 5b), which resulted in the decrease of shunt resistance and an increase of hole mobility. The pi-pi stacking played no role in **9** as confirmed from two-dimensional grazing incidence X-ray diffraction (2D-GIXRD) (Figure 5c) and hence, hydrogen bonding was solely responsible for the improvement in PCE.

**Figure 6:**
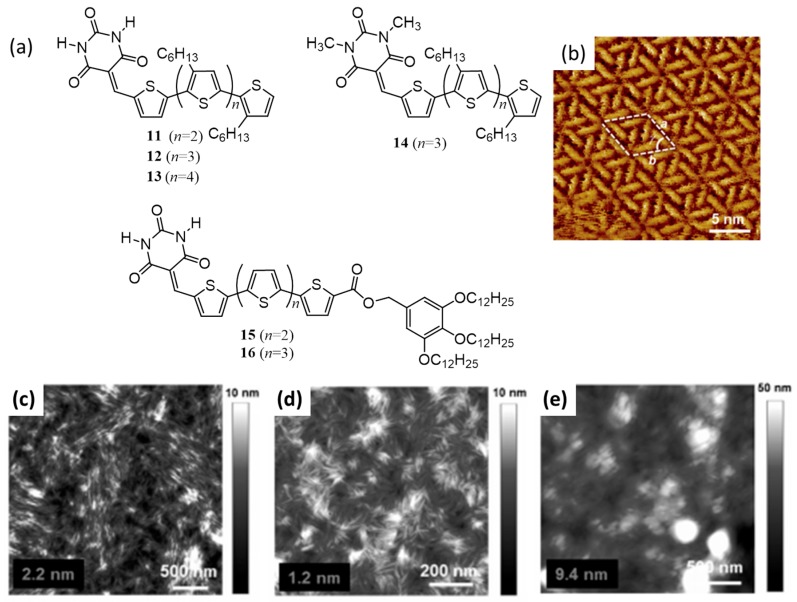
(**a**) Chemical structure of barbiturate-conjugated thienyl-[oligo(hexylthiophene)]s (**11**–**16**) synthesized by Yagai et al. [53]. (**b**) STM image of **13** on the 1-phenyloctane-highly oriented pyrolytic graphite (HOPG) interface. AFM topography images of the 1:1 (*w*/*w*) blend films of (**c**) **11**/PC_61_BM; (**d**) **12**/PC_61_BM and (**e**) **13**/PC_61_BM after annealing at 80 °C. The values at the bottom-left corner are root-mean-square (RMS) roughness. Adapted with permission from Ref. [53] Copyright 2014 WILEY-VCH Verlag GmbH & Co., KGaA.

It was well established that H-bond assisted self-assembly of barbiturate-conjugated pi-systems has the ability to form supramolecular rosettes, which on further stacking form highly ordered nanorods. Utilizing this idea, Yagai et al. in 2014, reported the self-organization and photovoltaic properties of barbiturate-conjugated thienyl-[oligo(hexylthiophene)]s derivatives (**11**–**13**; Figure 6a) [53]. The STM images of the molecules at the liquid-solid interface of 1-phenyloctane and highly oriented pyrolytic graphite (HOPG) revealed well-resolved two-dimensionally organized rosettes consist of six units aggregated by multiple H-bonds (STM image of **13** is shown in Figure 6b). The rosettes on further self-assembly through pi-pi stacking results in highly ordered nanorods. As cast BHJ films consist of the nanorods and PC_61_BM showed PCE of 1.38%, 0.97% and 1.49% for **11**, **12** and **13**, respectively. Interestingly, the devices of **12** showed a remarkable improvement of the photovoltaic performance upon increasing the annealing temperature from 50 to 80 °C (PCE from 0.83% to 2.1%) due to the betterment in the film morphology (Figure 6d) when compared to that of **11** and **13** (Figure 6c,e, respectively). Further improvement in PCE of **12** (3.01%) was achieved by using PC_71_BM. To confirm that the design was appropriate, the authors made derivatives with a non-hydrogen bonded reference molecule (**14**) and derivatives possess long aliphatic chains (**15**, **16**). The performances of these molecules were far less than that of the other three derivatives.

**Figure 7:**
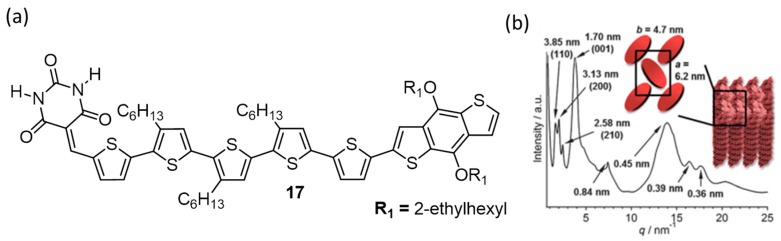
(**a**) Chemical structure of **17** synthesized by Yagai et al. [54]. (**b**) PXRD patterns of bulk samples of **17**/PC_61_BM at 25 °C. Adapted with permission from Ref. [54] Copyright 2016 The Royal Society of Chemistry.

The same group has reported functionalization of the best performing molecule (**12**) by BDT (benzo[1,2-*b*:4,5-*b*′]dithiophene) unit (**17**; Figure 7a) for further improving the photovoltaic device performance [54]. Upon fabrication of BHJ solar cells using a blend of **17** as donor and PC_61_BM as an acceptor (1:1) yielded a PCE of 2.98% without any thermal treatment. The thermal treatment decreases the PCE in the case of **17**, while it improves in the case of **12**. Hence, it was clear that an optimum phase-separated morphology for **17** was already achieved through solution processing, as supported by powder X-ray diffraction (PXRD) pattern which showed a one-dimensional columnar architecture via the formation of hydrogen bonded rosettes in the presence of PC_61_BM (Figure 7b).

As described in the above reports, hydrogen bonding can be effectively used for improving the photocurrent efficiency of organic molecules. However, it can also be detrimental, as observed in several cases. Aggressive hydrogen bonding results in the formation of macro domains, which hinders the charge transport in the molecule leading to poor efficiency. A few such examples are discussed in the following section.

**Figure 8:**
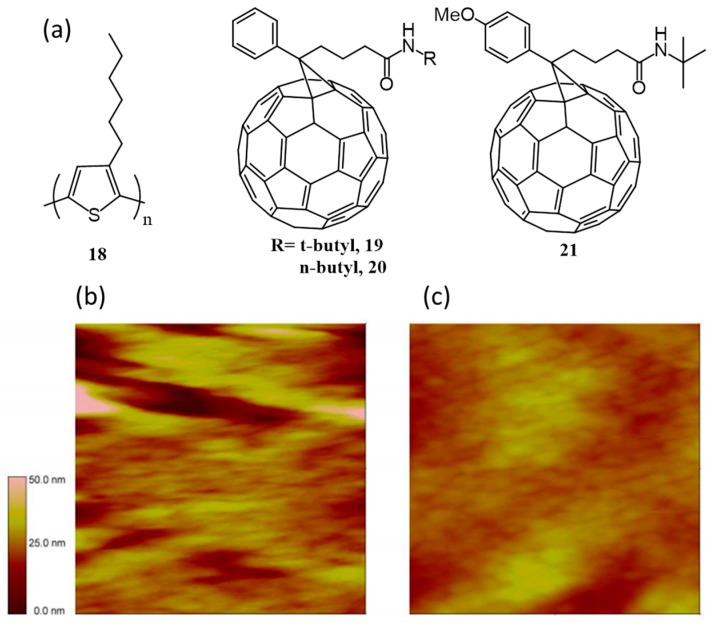
(**a**) Chemical structure of P3HT (**18**) and fullerene derivatives (**19**–**21**) synthesized by Li et al. [55]. AFM height images of cells fabricated from *o*-dichlorobenzene solution with the ratio of 1:4 of (**b**) **18**/**20** (**c**) **18**/**21** with 2.5% 1,12-diiodododecane by weight. Adapted with permission from Ref. [55] Copyright 2009 American Chemical Society.

In order to improve the phase separation of fullerene acceptor with a polymeric donor such as poly(3-hexylthiophene) or P3HT (**18**), Li et al. have designed and synthesized PC_61_BM analogues containing hydrogen bonding amide groups (**19**–**21**; Figure 8a) [55]. All the three derivatives were functionalized with amide group in the place of ester group of PC_61_BM, while in the case of **21** there is an additional methoxy group. Devices were made with **18**/fullerene derivative (1:4 *w*/*w*), and among the three, **21** gave an efficiency of 0.45%, while the reference PC_61_BM gave an efficiency of 0.42%. It was concluded that both fullerene derivatives have similar phase segregation scale. But upon annealing, amide fullerenes gave PCE almost zero, while PC_61_BM achieved an efficiency of 1.55%. As annealing was ineffective to amide fullerenes, the authors added 2.5% of 1,12-diiodododecane to the mixed solution of **18**/fullerenes. However, the efficiency of devices made from amide fullerene derivatives did not improve, while the performance of PC_61_BM based devices enhanced to 1.77% under similar conditions. AFM images of the active layer confirmed that 1,12-diiodododecane could not prevent the unfavorable aggregation nature of amide methanofullerene derivatives resulting in large phase separation (Figure 8b,c).

**Figure 9:**
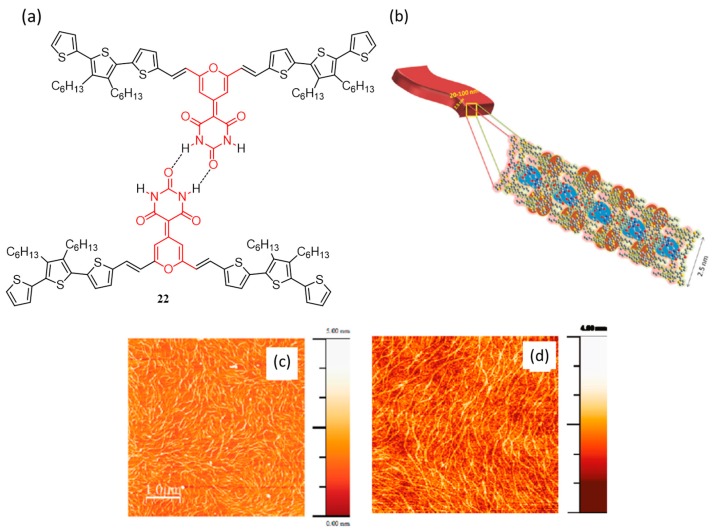
Schematic representation of (**a**) the intermolecular H-bonding between thiophene oligomers (**22**) synthesized by Patil et al. [56] and (**b**) the self-assembly of **22** into nanoribbons. AFM topography image of **22** in the (**c**) absence and (**d**) presence of PC_61_BM spin-cast from chlorobenzene solution. Adapted with permission from Ref. [56] Copyright 2011 American Chemical Society.

The effect of self-assembly induced by the cooperative hydrogen bonding and pi-pi stacking on the photophysical properties and the film morphology of an oligothiophene derivative (**22**; Figure 9a) was studied by Satish Patil et al. in 2011 [56]. The oligomer had a donor-acceptor-donor (D-A-D) structure with pyran-barbituric acid as the central core. H-bonding and pi-pi stacking interactions were induced by barbituric acid moiety and oligothiophene units, respectively. Due to the cooperative effect, the oligomer was able to form nanoribbons (Figure 9b) in the presence as well as in the absence of PC_61_BM as observed from AFM (Figure 9c,d, respectively). The authors have also made a non-hydrogen bonding model compound which did not form nanoribbons. The hole mobility of **22** was found to be ~7 × 10^−7^ cm^2^·V^−1^·s^−1^, measured using an organic field effect transistor (OFET) device architecture. The blend film of **22** with PC_61_BM (1:1 ratio) showed a maximum photovoltaic efficiency of 0.18%.

**Figure 10:**
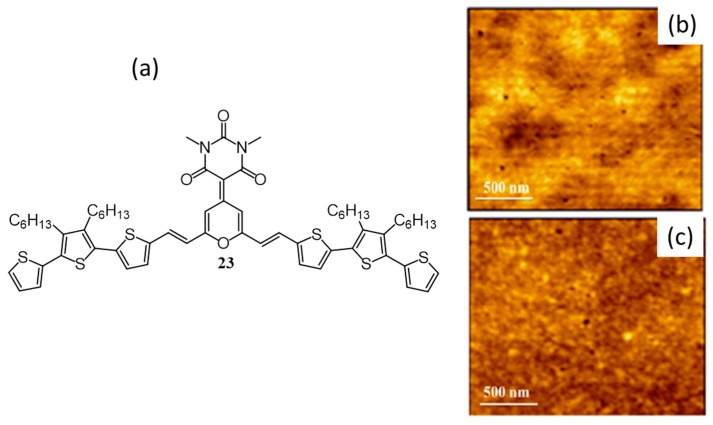
(**a**) Chemical structure of **23** synthesized by Patil et al. [57]. AFM images of **23** (**b**) without and (**c**) with PC_61_BM. Adapted with permission from Ref. [57] Copyright 2013 American Chemical Society.

The same group has synthesized another D-A-D type pi-conjugated oligomer (**23**, Figure 10a) with an aim to improve the solar cell efficiency [57]. As mentioned above, **22** consists of an acidic N–H proton and electronegative C=O that assist in self-assembling via hydrogen bonding. On the other hand, the hydrogen bonding is disrupted in the case of **23** by the introduction of a methyl group. Though **22** formed nanoribbons as mentioned above, films of **23** (pristine and blend) were homogeneous without any phase segregation (Figure 10b,c). Importantly, the photovoltaic device made from **23** gave an efficiency of 1.8%. The poor performance of **22** was attributed to the formation of nanoribbons which resulted in phase segregation with PC_61_BM. These results imply that prevention of H-bonding helped in developing better interdigitating networks which in turn enhanced the PCE. The reduced connection between the cathode and the active layer, due to an insulating layer formation by the reaction of acidic amide proton with calcium in the device was another reason for the poor performance of **22**.

**Figure 11:**
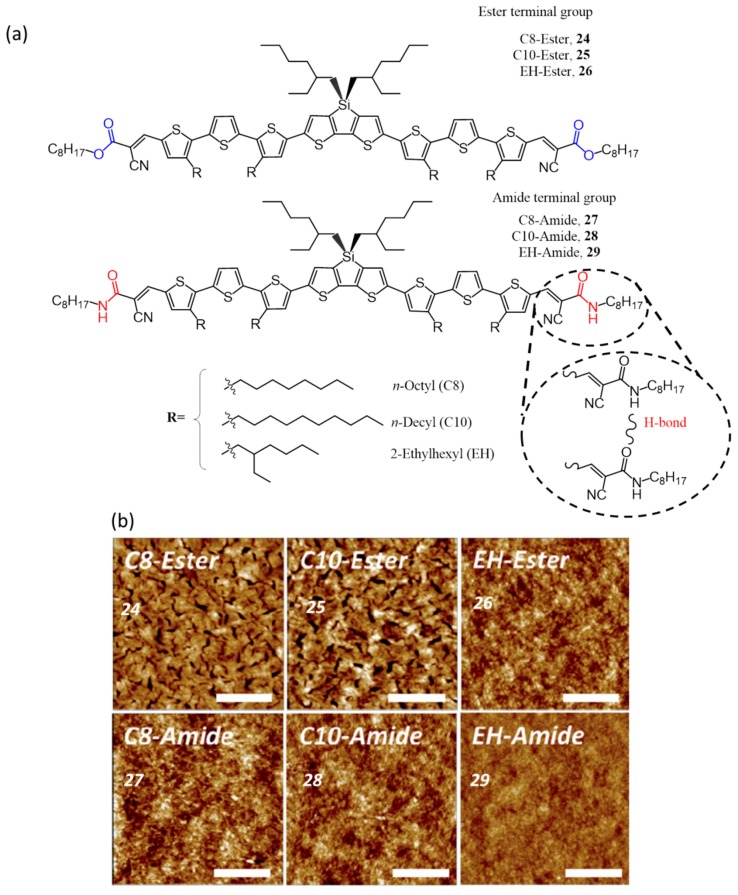
(**a**) Chemical structures of **24**–**29** synthesized by Kim et al. [58]. (**b**) AFM height images of the optimized active layer consisting of small molecule donors and PC_61_BM; the scale bar is 1 μm. The abbreviations of the authors are the following: **24**: C8-Ester, **25**: C10-Ester, **26**: EH-Ester, **27**: C8-Amide, **28**: C10-Amide and **29**: EH-Amide. Adapted with permission from Ref. [58] Copyright 2013 The Royal Society of Chemistry.

Another example to establish the fact that amide groups exhibiting strong H-bonding negatively affecting the solar cell efficiency was reported by Kim et al. They made six dithienosilole-based donors (**24**–**29**) consisting of different end groups (esters and amides) and alkyl side chains (*n*-octyl, *n*-decyl, and 2-ethylhexyl chains) as shown in Figure 11a [58]. Detailed studies showed that the optical properties of the molecules were affected by the alkyl side-chains as well as the end-functional groups. The absorption maximum of the thin films of the molecules was shifted bathochromically by 50–80 nm, due to the planarization of the conjugated backbone except in the case of **29** which showed 7 nm blue-shift. The effect of temperature on the charge carrier mobility properties of the molecules was studied at temperatures between 80 and 280 K using field-effect transistor device architecture. It revealed an increasing mobility trend in the order **25** > **24** > **28** > **27** > **26** > **29**. It was found that the H-bonding results a decrease in the planarity of the conjugated backbone, as a result, more energy was required for charge transport in amides when compared to esters. This poor charge transport property was well reflected in photovoltaic performance too; **29** exhibited extremely low efficiency (0.21%) among all, while **24** performed best and had a PCE of 4.31%. The PC_61_BM blend with esters (especially **24** and **25**) yielded good BHJ morphology consisting of small domain of 20–30 nm (RMS roughness of 2.7 nm). This contributed to the efficient separation of the excitons and facilitated the charge transport, leading to high PCEs. On the other hand, blends of **27** and **28** with PC_61_BM showed less distinct features in the morphology with much lower roughness values (~0.5 nm) due to the enhanced miscibility. Blend film of **29** lacks a clear interface, which resulted in the poor performance of the device. AFM images of all the blend films are shown in Figure 11b.

**Figure 12:**
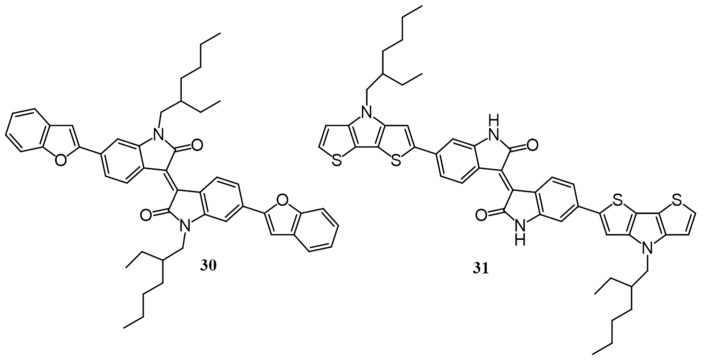
Chemical structure of **30** and **31** synthesized by Roncali et al. [59].

Molecules possessing free imide groups also have an adverse effect on the photovoltaic performance due to the formation of strong intermolecular hydrogen bonding as demonstrated by Roncali and co-workers [59]. Two donors **30** and **31** with a D-A-D structure involving isoindigo (IsI) as a central acceptor block and benzofuran (**30**) and dithienopyrrole (**31**) as a side donor unit were synthesized (Figure 12). The molecule **31** has a free imide group in the center and therefore, able to form intermolecular hydrogen bond while such an interaction is absent in the case of **30** as the nitrogen atom is alkylated. BHJ devices were prepared by spin-casting active films from a chlorobenzene solution of donor/PC_61_BM (1:2 *w*/*w*). **31** performed least (0.01%) as the hydrogen bonding locks the molecules into an unfavorable organization. However, planar heterojunction devices yielded PCE of 0.19% which decreased upon thermal annealing. On the other hand, **30** yielded a PCE of 0.15% which increased on annealing at 110 °C to 0.65%.

**Figure 13:**
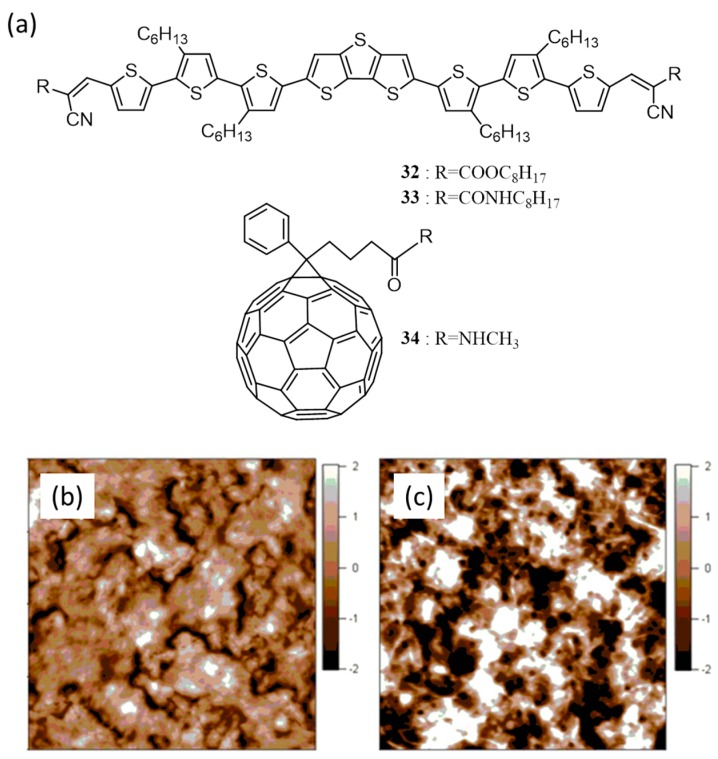
(**a**) The structure of the oligomers **32**–**34** synthesized by Wong et al. [60]. AFM height images of spin coated films from 10 mg·mL^−1^ chloroform solution of (**b**) **32** and (**c**) **33**. Adapted with permission from Ref. [60] Copyright 2014 Nature Publishing Group.

In 2014, Wong et al. reported the synthesis of two oligomers, one with ester (**32**) and the other with amide (**33**) end groups [60]. They synthesized a fullerene derivative with amide functionality (**34**) for comparison and also to investigate the utilization of hydrogen bonding self-assembly as a tool to control the morphology. The chemical structures of the oligomers and the fullerene derivative are shown in Figure 13a. UV-visible absorption studies revealed that the absorption maximum of **32** is more red-shifted than **33**. This was attributed to the presence of better electron withdrawing property of the ester group as compared to the amide. An amide hydrogen bonding in the case of **33**, disrupting the close pi-pi stacking between the planar conjugated back bone of the oligomer was also contributed to this effect. The absence of pi-pi stacking in **33** was supported by gracing incidence wide angle X-ray scattering studies. The AFM images of the oligomers revealed that **33** were able to form a fibrous structure which is beneficial for charge transport in BHJ, Figure 13c. However, devices fabricated using **33** performed poor, mainly due to unfavorable morphology induced by inter-molecular hydrogen bonding. Hole mobility calculated by space charge limited current (SCLC) method for **32** and **33** was found to be 6.3 × 10^−6^ and 1.1 × 10^−6^ cm^2^·V^−1^·s^−1^, respectively. BHJ devices fabricated with PC_71_BM and **32** (1:2) gave an efficiency of 1.15%, whereas, PC_71_BM and **33** (1:4) gave an efficiency of 0.40%. Stronger aggregation and large-scale phase separation were seen with **34** as the acceptor resulting in much lower efficiencies.

Pi-pi stacking is one of the most important interactions utilized for making ordered chromophore assemblies for optoelectronic applications. Effective pi-stacking is necessary for the efficient charge transport in organic materials and hence it plays a crucial role in photovoltaic device performances too. In the following section, we describe a few systems used in BHJ devices, which self-assemble mainly through pi-pi stacking.

**Figure 14:**
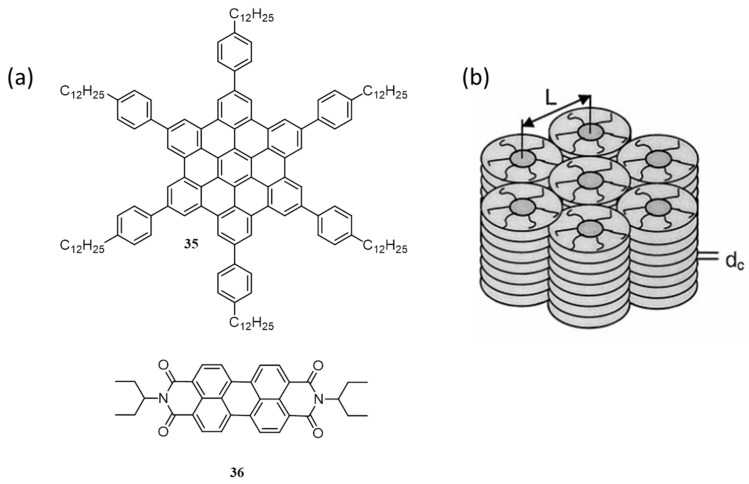
Chemical structures of **35** and **36** synthesized by Müllen et al. [61] and Schön et al. [62] respectively; (**b**) A simplified representation of the pi-pi stacking of the discotic molecules, where, L is the intercolumnar distance and d_c_ is the co-facial distance. Adapted with permission from Ref. [63] Copyright 2001 The American Association for the Advancement of Science.

Discotic liquid crystalline organic molecules exhibit intermolecular pi-pi stacking and mesoscopic long-range ordering. As a result, they show unique and interesting optoelectronic properties and can be used for advanced device applications. MacKenzie and co-workers have used a hexaphenyl-substituted hexabenzocoronene (**35**) [61] based discotic liquid crystalline molecule in combination with *N*,*N*′-*bis*(1-ethylpropyl)-3,4,9,10-perylenebis(dicarboximide) (**36**) [62]. The blended thin films of these molecules consist of vertically segregated perylene and hexabenzocoronene with the remarkably high interfacial surface area [63]. This is the first example of the use of self-assembling small molecule in organic photovoltaics. Figure 14a shows the chemical structure of the molecules and Figure 14b shows a simplified scheme of the pi-pi stacked assembly of **35**. The liquid crystalline ordering of **35** has micro-scale domain structures confirmed by polarized optical microscopy. Studies revealed the efficient photoinduced charge transfer between hexabenzocoronene and perylene. Due to this phenomenon, the blend film of **35**/**36** (40:60) showed photovoltaic response with external quantum efficiency >34% near 490 nm. The photovoltaic device under a light beam of 490 nm illumination at 0.47 mW·cm^−2^ gave a PCE of 2.0%.

**Figure 15:**
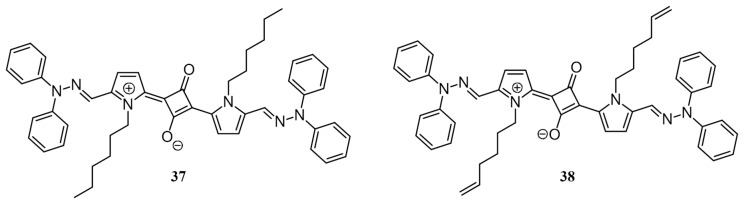
Chemical structures of **37** and **38** synthesized by Facchetti et al. [64].

Facchetti et al. studied how subtle pi-core substituent variations affect photovoltaic responses of functional chromophoric systems [64]. In 2010, they have reported the synthesis of two new squaraine dyes, substituted at the pyrrolic nitrogen with *n*-hexyl (**37**) or *n*-hexenyl (**38**) chains (Figure 15). The dyes exhibited a remarkable difference in optoelectronic properties due to the variation in packing as evident from the crystal structure. The crystal of **38** exhibits shorter interplanar and the interstack distance between cores (3.138 and 7.343 Å, respectively) in comparison to **37** (3.417 and 7.809 Å, respectively). The crystal packing of **38** also features edge-pi interaction between the terminal hexenyl double bond and phenyl rings (2.85–2.90 Å), while no such interaction was present in **37**. Upon fabrication of BHJ solar cells using conventional architecture, **38** exhibited a maximum PCE of 2.05%, while **37** had a maximum PCE of 1.47%. This was attributed to the presence of more compact and denser nanofibrils of **38** due to the non-covalent alkenyl-phenyl interaction.

**Figure 16:**
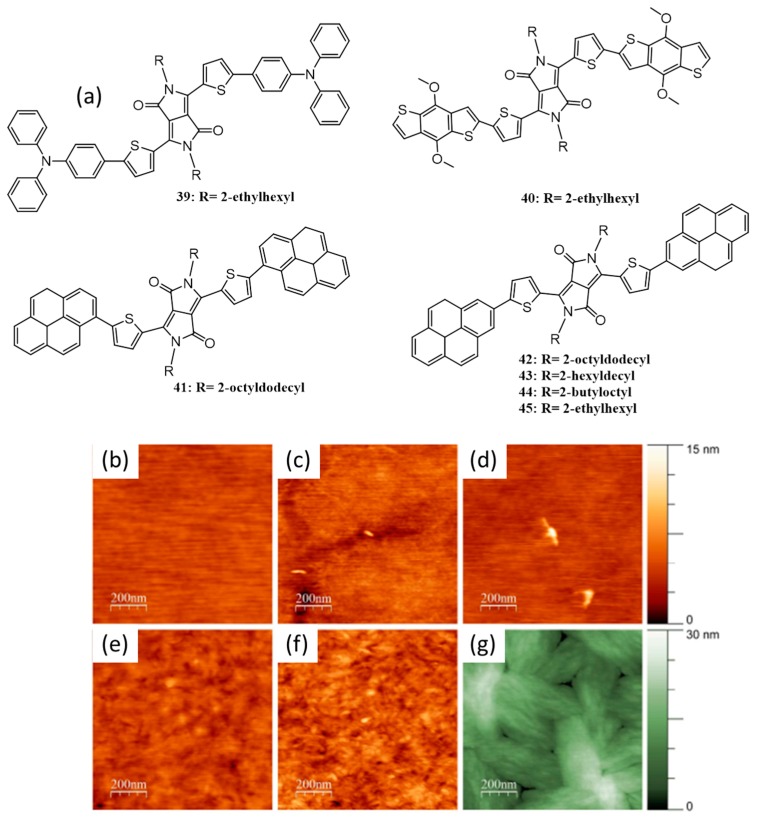
(**a**) Chemical structures of **39**–**45** synthesized by Fréchet et al. [65]. Tapping-mode AFM images of the blends of (**b**) **39**; (**c**) **40**; (**d**) **41**; (**e**) **42**; (**f**) **43**; (**g**) **44** with PC_71_BM. Adapted with permission from Ref. [65] Copyright 2011 WILEY-VCH Verlag GmbH & Co., KGaA, Weinheim.

The incorporation of pi-stacking moieties as end groups would facilitate small molecules/oligomers to stack efficiently, leading to the enhancement in charge carrier mobility properties. In 2011, Fréchet and coworkers explored this idea by preparing seven small molecules consisting of diketopyrrolopyrrole (DPP) as the electron-deficient core [65]. DPP is known to provide solution-processability and film-state molecular ordering. The molecules were end-functionalized with various acceptors such as triphenylamine (**39**), BDT (**40**), and pyrene (**41**). Further, the effect of end-group symmetry was studied by preparing **42**, where the regio-connectivity between pyrene and the chromophoric core was changed. To study the effect of alkyl chains on molecular packing and solar cell efficiency, the DPP unit of **42** was incorporated with 2-hexyldecyl (**43**), 2-butyloctyl (**44**), and 2-ethylhexyl (**45**). All the chemical structures of the oligomers are shown in Figure 16a. The blend film of **43** with PC_71_BM gave the highest photocurrent efficiency of 4.1%, whereas, lower efficiency was obtained for **39** (1.3%), **40** (1.7%), **41** (0.7%), **42** (2.7%) and **44** (3.0%). The molecule **45** had poor solubility for processing it into a device and hence its photovoltaic properties were not investigated. To understand the reason for the variation in photovoltaic performances of the molecules, the active layer morphology for all derivatives were studied by AFM (Figure 16b–g). The presence of relatively crystalline domains with sizes of ~10–30 nm was assumed for the better performances of **43** and **44**.

**Figure 17:**
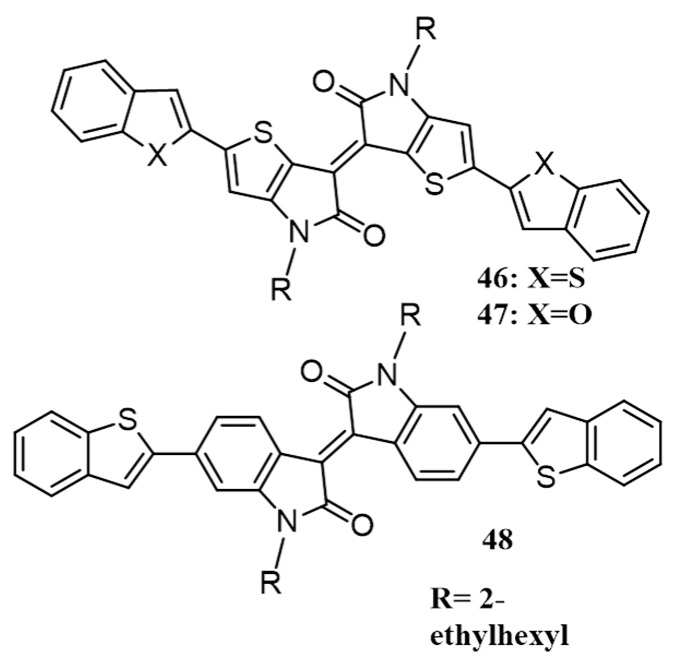
The chemical structures of **46**–**48** synthesized by Mori et al. [67].

Thienoisoindigo and isoindigo based copolymers have been known to exhibit balanced ambipolar charge transport properties with both electron and hole mobilities exceeding 0.1 cm^2^·V^−1^·s^−1^ [66]. Considering this aspect, Mori and co-workers have synthesized and investigated the photovoltaic performance of two molecules consisting of thienoisoindigo core end-capped with benzothiophene (**46**), and benzofuran (**47**), and another molecule with isoindigo core end capped with benzothiophene (**48**; Figure 17) [67]. The electronic structures of the molecules were studied by density functional theory (DFT) calculations. It revealed that the optimized molecular structures of **46** and **47** are almost flat, while **48** is non-planar because of the twist between benzothiophene and isoindigo units (23.9°) as well as the bent of isoindigo unit (11.3°). As a result, the intermolecular pi-pi stacking was stronger in **46** and **47** resulting in higher crystallization temperature (260–270 °C) than that of **48** (220 °C). In accordance with these observations, the charge-carrier mobilities of **46** and **47** are two-three order of magnitude higher (4.0 × 10^−7^ and 2.1 × 10^−6^ cm^2^·V^−1^·s^−1^, respectively) than that of **48** (1.9 × 10^−9^ cm^2^·V^−1^·s^−1^) as measured by SCLC method. BHJ devices of **46** and **47** with PC_71_BM showed good photovoltaic device efficiencies of 2.4% and 1.5%, respectively. On the other hand, devices made of **48** under identical conditions did not work. The planar structure of the thienoisoindigo unit, therefore, has an obvious effect on the molecular packing properties and the thin-film morphology. This unit also decreases the steric interference caused by the benzothiophene and benzofuran unit thereby improving the photovoltaic properties.

**Figure 18:**
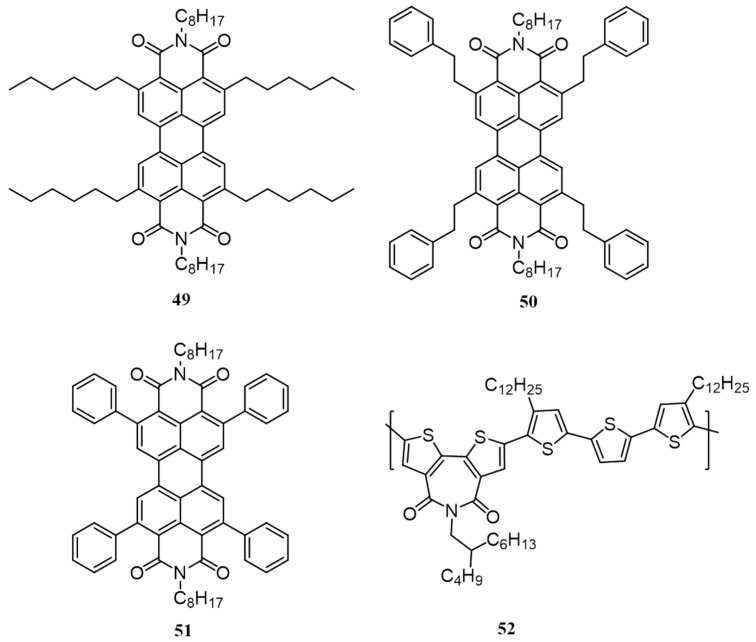
The chemical structures of **49**–**52** synthesized by Wasielewski et al. [74].

Due to the low-lying LUMO and high electron mobilities, perylenediimide (PDI) derivatives have been extensively used as an acceptor in OSCs for more than a decade [68,69]. However, PDIs give low PCE values because of the poor BHJ morphology. They have a strong tendency to stack through the extended pi-surfaces and resulting in large crystallites and macrophase separation in the bulk active layer. Moreover, PDI derivatives easily form excimers which act as traps for electrons thus reducing the overall efficiency. In order to get rid of these issues, twisted PDI dimers have been used by connecting the imide position through a hydrazine linker or by functionalizing the “bay positions”. Incorporation of bulky “swallow tail” PDI side chains at the imide position was also found to introduce twisting in the molecular packing [70,71,72]. Although adopting twisted PDIs have led to very high efficiencies, these too suffer from certain limitations. Completely preventing pi-pi stacking is not a possible solution to inhibit excimer formation since an absence of continuous pathway can lead to charge recombination. In this context, Wasielewski et al. in 2013 introduced a new method to inhibit the excimer formation by substituting suitably at C2, C5, C8, and C11 positions of PDI [73]. This arranges the molecules in a slip-stacked manner thereby inhibiting excimer formation without rupturing the pi-stacking interactions. This novel idea was then used to develop BHJ solar cells for which they synthesized three PDI derivatives **49**, **50** and **51** with hexyl, phenethyl and phenyl side chains, respectively as acceptors and **52** as the donor polymer (Figure 18) [74]. Upon fabrication of the inverted solar cell, the blend of **51**:**52** gave the highest efficiency of 3.64%, while blends of **49** and **50** with **52** gave an efficiency of 0.65% and 1.20%, respectively. From the AFM analysis of the active layer, it was clear that the improved efficiency in the case of **51** was mainly due to moderate crystallinity and formation of micrometer-sized crystallites, while **49** forms the roughest and **50** forms the smoothest films.

**Figure 19:**
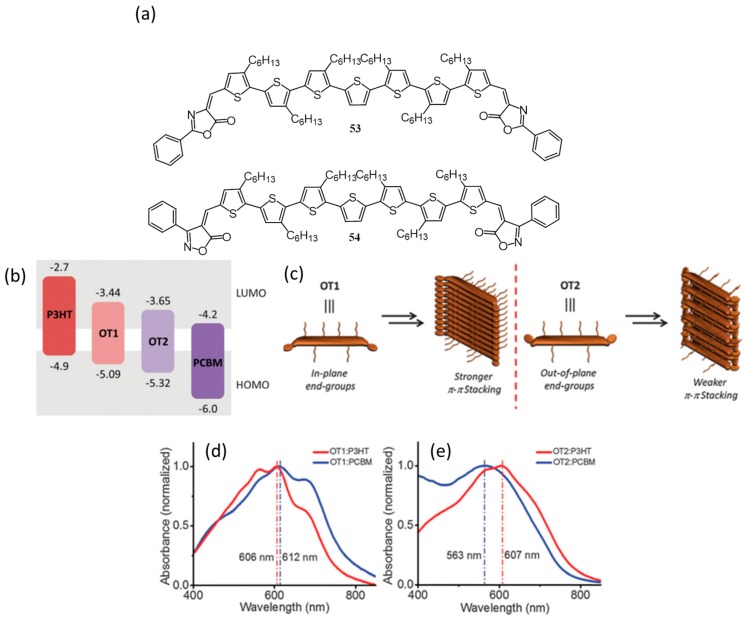
(**a**) The chemical structure of oligomers **53** and **54** as synthesized by Nair et al. [75]. (**b**) Scheme showing the comparison of the HOMO–LUMO levels of the oligomers with that of **18** and PC_61_BM (**c**) Simplified schematic representation of the supramolecular organization of **53** and **54** in the self-assembled state. Normalized absorption spectra of (**d**) **53** and (**e**) 5**4** blend films (1:1 *w*/*w*) with **18** or PC_61_BM spin-cast from chlorobenzene solution. Abbreviated by authors as following—**53**: OT1 and **54**: OT2.

In 2015, our group reported the optical, electronic and photovoltaic properties of thiophene oligomers end-functionalized with oxazolone (**53**) and isoxazolone (**54**) derivatives (Figure 19a) [75]. We have illustrated that small structural changes significantly affect the molecular and supramolecular properties of the oligomers. More importantly, it also affects the p/n polarity of the molecules in a bulk-heterojunction device architecture. Comparison of the highest occupied molecular orbital (HOMO) and lowest unoccupied molecular orbital (LUMO) levels of **53** and **54** with that of PC_61_BM and **18** (P3HT) revealed that, in principle, both of them can act as donors to PC_61_BM and acceptors to **18**. However, **53** acted as a p-type material (PCE of 0.75% with 1:1 *w*/*w* PC_61_BM), whereas, **54** behaved more like an n-type material (PCE of 0.19% with 1:1 *w*/*w* **18**) in BHJ solar cells with an inverted configuration. This observation could be explained by considering the molecular (energy levels) and supramolecular (film state packing) properties of the molecules. The shallower HOMO and LUMO of **53** (compared to **54**) made it better electron donor to PC_61_BM, whereas, deeper HOMO and LUMO of **54** are helpful for accepting electrons from **18** (Figure 19b). Depicted by DFT, XRD, thermogravimetry analysis (TGA) and UV-vis absorption studies, **53** has a coplanar structure, whereas, **54** consists of an out-of-plane isoxazolone acceptor. As a result, the former forms highly ordered, stable aggregates through strong pi-pi stacking and latter exhibit weaker pi-pi stacking. Supramolecular organization of **53** and **54** in the self-assembled state is schematically depicted in Figure 19c. Detailed absorption studies proved that the self-assembly of **53** was intact upon the addition of **18** or PC_61_BM (Figure 19d). In the case of **54**, the addition of PC_61_BM destroys the assembly resulting in a hypsochromic shift of the absorption maximum (Figure 19e). The p/n polarity of the oligomers was also confirmed by flash-photolysis time-resolved microwave conductivity analysis.

**Figure 20:**
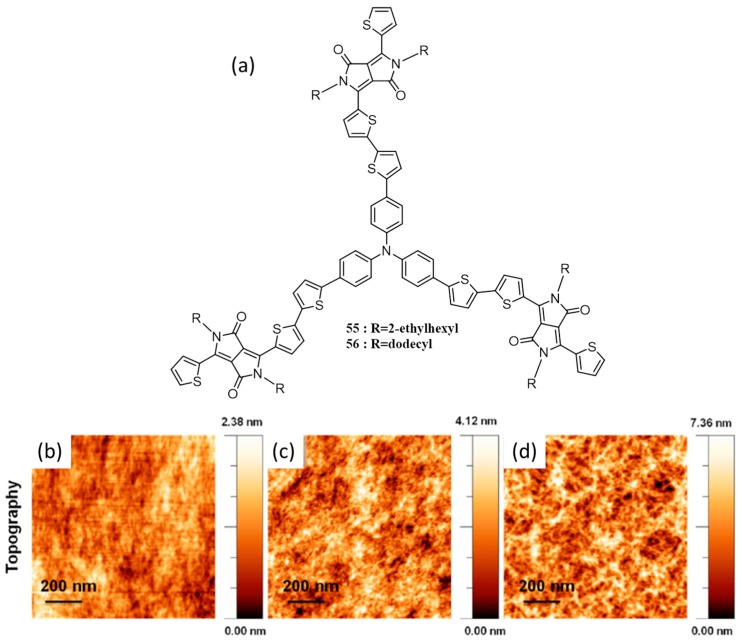
(**a**) The chemical structures of **55** and **56** synthesized by Stupp et al. [78]. AFM topography images of (**b**) **55** cast from chloroform; (**c**) **56** cast from chloroform; and (**d**) **56** cast from chlorobenzene. Adapted with permission from Ref. [78] Copyright 2016 American Chemical Society.

Several “star-shaped” donors with complex architectures (C-3 symmetric or tripodal) capable of forming self-assembled structures have been reported for photovoltaic applications with efficiencies surpassing 4% [76,77]. Stupp and co-workers have foreseen that the increased pi-pi stacking interactions in disc-like molecules would facilitate the formation of self-assembled fibers when appended with solubilizing alkyl side chains [78]. Very recently, they have designed and synthesized tripodal donor molecules, **55** (with 2-ethylhexyl) and **56** (with dodecyl), where electron-donating triphenylamine functions as the donor core and DPP units flanked by thiophene groups chosen as the chromophoric “arms” (Figure 20a). The films of the active layer were first investigated by AFM and 2D-GIXRD. **55** forms featureless films (Figure 20b) while **56** forms short nanowires when casting from chloroform solvent (Figure 20c). The self-assembly of **56** was further enhanced when casting from chlorobenzene while no enhancement in morphology was seen for **55** (Figure 20d). GIXRD results showed that **56** have a tendency to self-assemble with cofacial stacking to a greater extent than **55**, which was well supported by the classical molecular dynamics simulation studies. AFM analysis showed that **56** forms small nanowires in chloroform, but forms featureless films after blending with PC_71_BM. This was attributed to two reasons; PC_71_BM disrupt the stacking of the donor and the use of highly volatile solvent chloroform did not provide the molecules enough time to stack efficiently. A combination of m-cresol and DIO promotes assembly of **56** into a well-dispersed network of nanowires, while the use of this solvent combination has increased the roughness in the case of **55**. Solar cells were fabricated containing **55** and **56** using optimized weight ratios of donor/PC_71_BM of 1:3 and 1:2, respectively. Without any solvent additives, devices of **55** show higher performance than those of **56**, with PCEs of 2.41% and 1.29%, respectively. With the addition of DIO, a significant improvement in the PCE (4.19%) was observed for **56**, whereas, only marginal increase was observed for **55** (2.75%) under identical conditions. The performance of the molecules was further improved by the addition of 1.0 vol % of m-cresol as a solvent additive, which mainly functions as a dispersant. The fully optimized devices have a PCE of 4.39% for **56** and 2.91% for **55**. The authors concluded that the use of linear alkyl chains rather than branched ones boosts supramolecular self-assembly in tripodal systems via pi-pi stacking, thereby bestowing a 50% improvement in device performance. In other words, the supramolecular assembly of **56** with linear alkyl chains creates ordered aggregates, while steric effects due to branched alkyl chain prevent **55** from stacking, thus forming amorphous domains with more structural defects that contribute to trap-mediated recombination.

**Figure 21:**
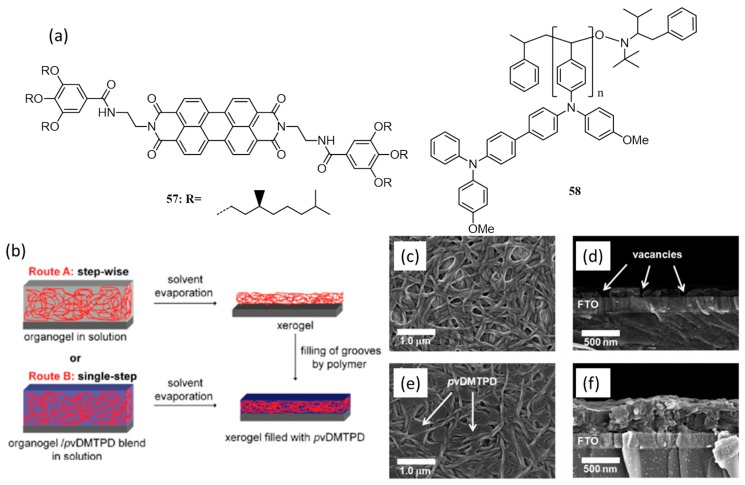
(**a**) Chemical structures of **57** and **58** synthesized by Würthner et al. [79] and Thelakkat et al. [80] (**b**) Schematic representation of the blending of organogel and polymer via route A and route B. SEM images of (**c**) **57**-xerogel via route A and (**d**) corresponding cross section; (**e**) Surface image of **57**-xerogel/**58** blend (3:1) via route B and (**f**) corresponding cross-section. Abbreviated by authors as **58**: poly(vinyl-dimethoxytetraphenylbenzidine) (*pv*DMTPD). Adapted with permission from Ref. [81] Copyright 2009 American Chemical Society.

The synergistic effects of hydrogen bonding and pi-pi stacking have been utilized in organic molecules for solar cell applications. In 2009, research groups of Würthner and Thelakkat made a nanostructured donor-acceptor interface of an n-type organogelator (**57**) with a p-type polymer (**58**) by combining hydrogen bonding and pi-pi stacking interactions, Figure 21a [79,80,81]. The nanostructures were prepared via two routes (Figure 21b); the first method (route A) involves the preparation of the organogel of **57** followed by removal of the solvent. The pores and grooves of the resulting xerogel were subsequently filled by the polymer, **58**. The second method (route B) is a single-step process consisting of the blending of **57** and **58** yielding a three-dimensional network of the organogel of former embedded into an amorphous matrix of latter. In route B, blends of **57**/**58** were taken in three different ratios of 3:1, 1:1, and 1:3. The composition 3:1 delivered the highest current and power conversion values. The film morphology obtained via both routes was examined by SEM (Figure 21c–f), which showed the superstructure of **57**-xerogel obtained via route A is highly entangled and aggregated. However, in route B, the amorphous polymer **58** occupies the vacancies in the xerogel of the blend film. The presence of polymer also prevents the formation of thicker gel strands resulting in high donor-acceptor interface. Films prepared by both methods were smoothened by doctor blading the surface using the polymer in chlorobenzene solution. To understand the importance of gelated network structure on charge separation and transport, devices were made using a non-gelating derivative of **57**, *N*-(1-nonyldecyl)-*N*′-(1-pentylhexyl)-perylene-3,4,9,10-tetracarboxylic bisimide and measured its photovoltaic properties in combination with **58**. This device gave an efficiency of 0.003%, while the xerogel sample gave the highest PCE of 0.04% via route B.

**Figure 22:**
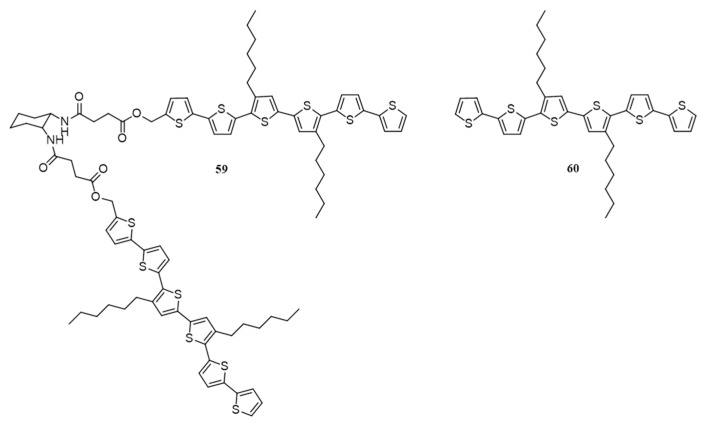
Chemical structures of **59** and **60** synthesized by Stupp et al. [83].

Hairpin molecules have the ability to form one-dimensional (1D) nanowires through pi-pi stacking and hydrogen bonding. These wires, with a grooved exterior, are known to provide high interfacial contact between donor and acceptor components [82]. Stupp et al. have synthesized a hairpin shaped gelator by connecting 1,2-cyclohexyldiamine having hydrogen bonding groups to a sexithiophene (**59**) self-assemble into 1D nanowires, Figure 22 [83]. The packing of the molecules will result in grooved nanostructures with void space, where small molecules like PC_61_BM get incorporated easily. At concentrations >0.5 wt %, the interpenetrated nanowires lead to the formation of gels in solvents such as toluene and chlorocyclohexane. Photovoltaic devices of **59** and PC_61_BM (40:60) in chlorobenzene gave the highest efficiency of 0.22% upon annealing at 80 °C, which decreased a little at 90 °C and drastically at 100 °C. Differential scanning calorimetry and optical polarizing microscope analysis suggested that the nanostructures melt at 109 °C and do not reassemble upon cooling. This irreversible change of **59** in higher temperature is attributed to the drop in efficiency. They later changed the acceptor to PC_71_BM, obtained an efficiency of 0.48% at 90 °C. The role of self-assembly in this system was studied by fabricating devices with dihexylsexithiophene (**60**). This linear molecule consists of the same oligothiophene moiety but devoid of hydrogen bonding units. The PCE of the devices without annealing was 0.32%, which was similar to the **59**/PC_71_BM devices. Upon annealing at 80 °C, the efficiency increased to 0.39% but further increase in temperatures drastically decreased the efficiency as in the case of **59**. However, the mean PCE of **59** was 0.42%, whereas, the mean PCE of **60** was 0.36%. Welch’s unpaired *t*-test at the 1% significance level proved the difference in mean between **59** and **60** was statistically significant. This work signifies the importance of grooved structure, which promotes receptor-ligand type configurations in bulk heterojunctions and improves device efficiency.

**Figure 23:**
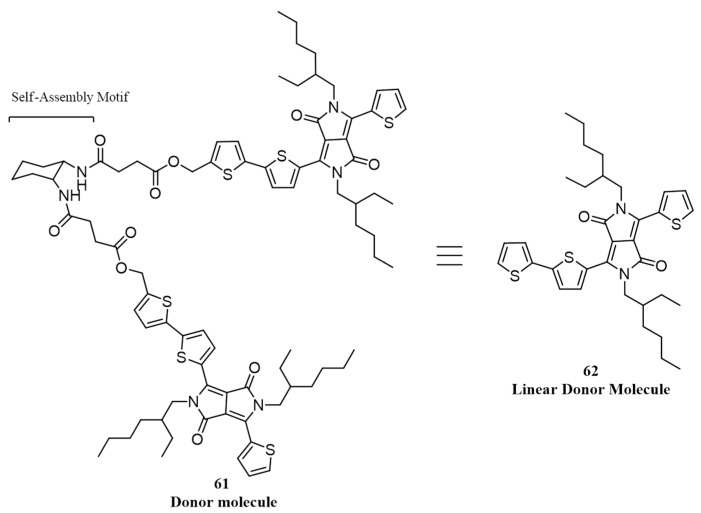
Chemical structures of **61** and **62** synthesized by Stupp et al. [84].

The same group has reported another hairpin-shaped molecule consisting of *trans*-1,2-diamidocyclohexane as core and DPP as the arm (**61**) having a chemical structure given in Figure 23 [84]. Self-assembled nanowires were prepared through a step-wise cooling process with minimal stirring of solutions. The fabricated devices of the nanowires with PC_71_BM (1:1 wt %) as acceptor showed an optimized efficiency up to 0.53%, which was significantly higher than those of devices prepared by simple mixing of donor and acceptor molecules. For comparison purposes, the authors have made cells using a linear molecule that contains the same arm of the hairpin molecule (**62**), which gave an efficiency of only 0.24%. Therefore, synergistic action of hydrogen bonds and pi-pi stacking in donor molecules that created long nanowires by self-assembly results in devices that are more efficient than those built with analogous donor molecules containing only the conjugated structure.

**Figure 24:**
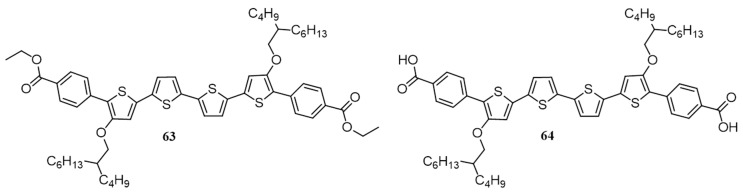
Chemical structures of the quarterthiophene core with diester (**63**) and diacid (**64**) end-groups, synthesized by Lam et al. [85].

Dicarboxylic acid is an excellent end-functional group for enhancing intermolecular connectivity and crystallinity in solution-processable conjugated small molecules. As a proof of concept, quarterthiophenes end-functionalized with diester (**63**) and diacid (**64**) groups were synthesized by Lam and co-workers (Figure 24) and studied their photovoltaic properties [85]. The idea of using an acid end group is that it may result in strong hydrogen bonding which may assist pi-pi stacking between the oligomers. Consequently, the formation of a pseudo-polymer may happen, which was expected to improve the charge transport among the chains and enhance the efficiency of diacid in comparison with diester. In the film state absorption studies, an extended red-shift absorption in the visible region was observed in **64** with reference to **63**, which is an indication of hydrogen bond induced pi-pi stacking in the molecule. The enhanced pi-pi stacking was also evident in the thermogravimetric analysis studies as **64** exhibited higher thermal transition temperatures than that of **63**. Solar cells were fabricated using **63** and **64** as donors and PC_61_BM as acceptor. **63** exhibited an efficiency of 0.39% for films fabricated from chlorobenzene and 0.42% for films from *o*-dichlorobenzene. For **64**, a solvent mixture of THF and DMF was used as the molecule has poor solubility in chlorinated solvents, and the PCE was 0.91%.

**Figure 25:**
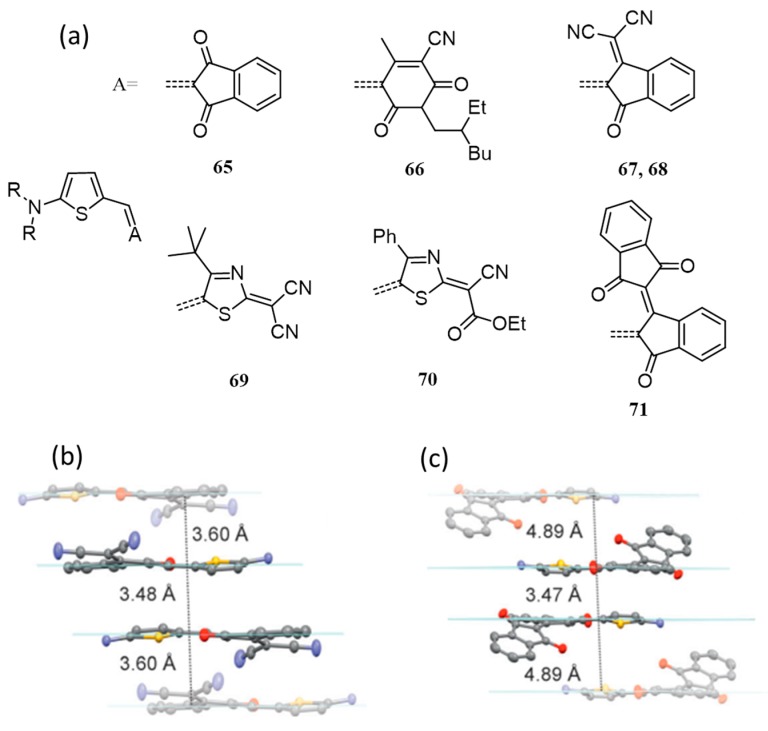
(**a**) Molecular structures of the investigated D–A dyes (**65**–**71**), synthesized by Würthner et al. [89].The substituents are R = *n*Bu and R′ = Et for **68** and R = R′ = *n*Bu for all other dyes. Adapted with permission from Ref. [89] Copyright 2011 WILEY-VCH Verlag GmbH & Co. KGaA, Weinheim.

Dipole-dipole interaction is another supramolecular force successfully used for developing BHJ based organic solar cells. According to the Bassler model, increased energetic disorder associated with dipole moments is detrimental to charge hopping and hence molecules with minimum or zero dipole moments are considered promising for optoelectronic applications [86,87]. However, certain reports suggest that the self-assembled centrosymmetric dimer formation of highly dipolar D–A systems eliminate molecular dipole moments at the supramolecular levels [88]. To illustrate the use of highly dipolar D–A substituted pi-systems in BHJ solar cells, Würthner and co-workers have synthesized six D–A dyes, **65**–**71** (Figure 25a) [89]. Among them, **66**, **69** and **70** possess large ground state dipole moment (μ_g_; 12–13 D) and show small changes of the dipole moment (Δμ) upon optical excitation, whereas, **65**, **67**, **68** and **71** exhibits distinctly lower μ_g_ and higher Δμ values. BHJ photovoltaic devices were fabricated with a device structure of ITO/PEDOT:PSS(40 nm)/dye:PC_61_BM/Al (120 nm). Most of the cells showed low efficiency, but devices prepared from **67** and **68** exhibited higher *J*_SC_ and PCEs of 2.3% and 3.0%, respectively. Cells were later optimized using **68**, where clear improvements were seen upon replacing PC_61_BM with PC_71_BM. Replacement of hole-transporting layer, PEDOT:PSS by MoO_3_ has further improved the efficiency to 4.5% and 5.1% under standard and reduced lighting conditions, respectively.

**Figure 26:**
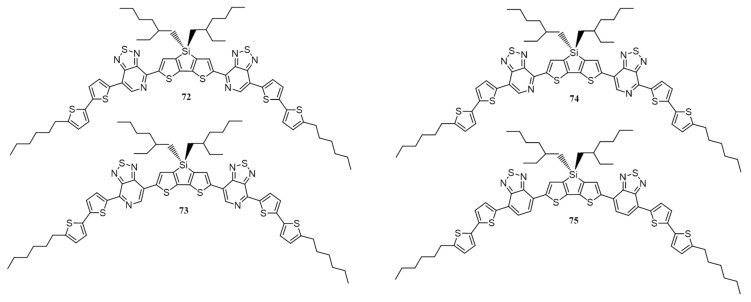
Molecular structures of compounds **72**–**75** synthesized by Heeger, Bazan et al. [91,92,93].

In 2012, Heeger, Bazan and co-workers synthesized four D′-A-D-A-D′ type isomorphic small molecules for investigation of dipole moment effect and conformational stability on the self-assembly and photovoltaic performance [90]. All the four molecules are isomeric to each other, and the only difference in the structures is the position of the nitrogen atom. Three of them are based on symmetrical and unsymmetrical regiochemistries of the acceptor, [1,2,5]thiadiazolo[3,4-*c*]pyridine (PT; **72**–**74**) [91,92,93]. **75** has 2,1,3-benzothiadiazole (BT) acceptor as the core (Figure 26). Though the structures of these molecules are quite similar but they exhibited a significant difference in PCE mainly attributed to the difference in the molecular packing. The symmetrical molecules **72** and **73** gave best efficiencies (with 0.25 *v*/*v* DIO), 6.70% and 5.56%, respectively, while unsymmetrical molecule showed lower efficiency (3.16%). The depletion in the efficiency of **74** can be attributed to the packing frustration that resulted from the unsymmetrical geometry. With the increase in the *v*% of DIO, the efficiency goes down for all the four molecules. The molecules **72** and **73** possess strongest Bragg’s reflections with scattering wave vector at ≈3 and ≈18 nm^−1^ which attributes to crystalline domains and pi-pi stacking, respectively. For both, an amorphous scattering was observed at ≈13 nm^−1^. For **74**, a broad peak was extended from 12 to 20 nm^−1^, which reflects its large degree of disorder. **75** exhibits lowest degree of order and HRTEM showed a lack of phase separation within the films, which resulted in low performance (0.19%). It was concluded that the net dipole moment resulted in the difference of self-assembly in PT and BT based molecules. In BT containing molecule, local dipole moment distribution frustrates the self-assembly process. On the other hand, in the case of the PT-based molecule, the pyridyl *N* atom imparts extra stability by minimizing the variation in net dipole moment and thereby enhancing the morphology of the active layer.

## 3. Polymers

Polymers are macromolecules composed of a large number of repeating units. In general, the organic polymers are insulating in nature due to the absence of free charge carriers. However, in 1977 Heeger, MacDiarmid and Shirakawa have changed the view on polymers as they could make polyacetylene conductive just like a metal through doping [94]. It was later understood that a key feature of a polymer that will be conductive is the presence of a linear series of overlapping *p_z_* orbitals with sp^2^ or sp hybridization (conjugated double or triple bonds), creating a chain of delocalized electrons. Consequently, the charge carriers are free to move along the long conjugated backbone, and hence they exhibit very interesting electronic properties. However, the electron delocalization locks in a planar conformation between monomers have often lead to rod-like behavior and liquid crystallinity in some polymers. This phenomenon drastically alters their behavior, often decreasing solubility and increasing kinetically trapped metastable structures. In self-assembling polymers, interpolymer charge transport is more difficult and normally limited by charge hopping from one polymer to the adjacent polymer. Therefore, these challenges needed to be solved by introducing supramolecular interactions within the polymers, thereby minimizing the death of charge carriers via charge hopping within them. Supramolecular interactions have been utilized in several non-conjugated polymers and conjugated polymers for developing BHJ solar cells. Typical examples in this category are discussed in the following section.

**Figure 27:**
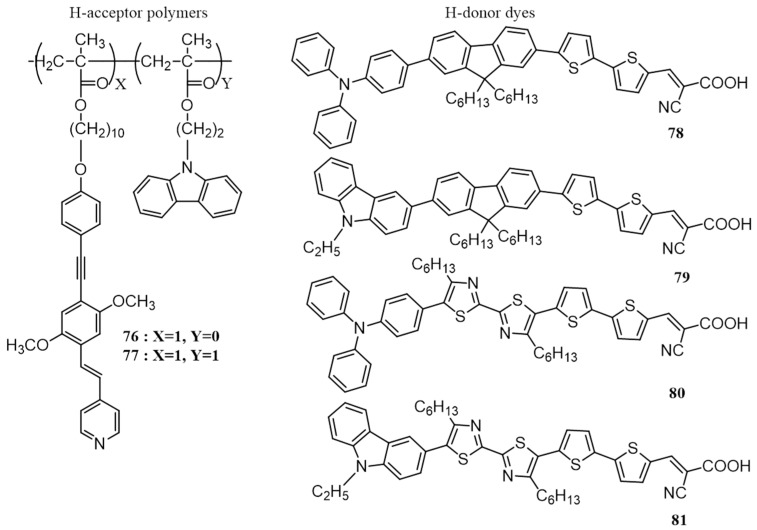
Chemical structures of the polymers (**76**,**77**) and low-bandgap molecules (**78**–**81**) synthesized by Lin et al. [95].

Construction of supramolecular side-chain polymers via association of proton acceptor (H-acceptor) polymers with low-bandgap proton donor (H-donor) dyes is a strategy adopted by many leading research groups for developing materials for photovoltaic applications. In 2009, Lin et al. prepared a number of such polymers by mixing pyridyl pendant containing H-acceptor polymers (**76**, **77**) with low-bandgap H-donor dyes bearing terminal cyanoacrylic acids (**78**–**81**) in a molar ratio of 1:1 (Figure 27) [95]. The preparation of the supramolecular polymer complexes involves the dissolution of the dyes and polymers in THF, evaporation of the excess solvent and finally drying in vacuum at 60 °C. The complexation of the dyes and the polymers through H-bonding occurred during the solvent evaporation process. The hydrogen bonding in supramolecular side-chain polymers was confirmed by Fourier transform infrared spectroscopy (FTIR). Bulk-heterojunction solar devices were fabricated using the H-bonded polymer complexes as donor and PC_61_BM as an acceptor in the wt ratio of 1:1. The highest efficiency (0.50%) was obtained for the active layer composed of **76**/**80**:PC_61_BM. The authors have also demonstrated the importance of H-bonding by making a device consisting of a physically blended active layer of **76** and a non-hydrogen bonding methyl ester derivative of **78**, which showed a lower efficiency of 0.16%.

**Figure 28:**
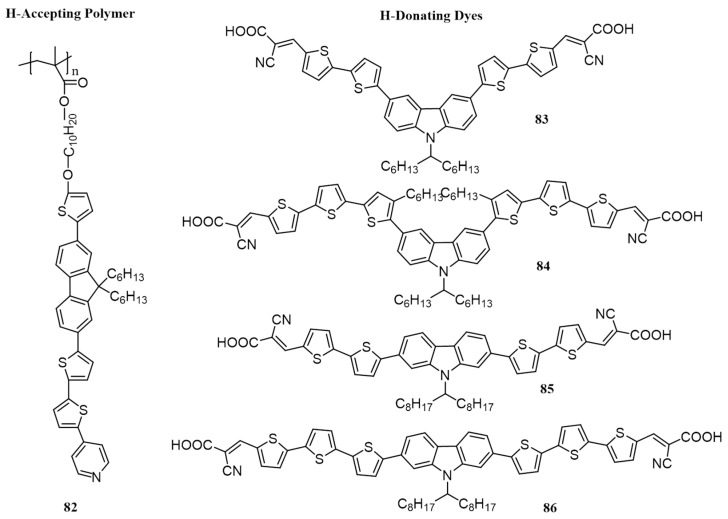
Chemical structures of the H-acceptor side-chain polymer (**82**) and H-donor dyes (**83**–**86**) synthesized by Lin et al. [96].

The same group reported a similar work in 2010, in which a series of H-bonded cross-linking polymers were prepared by complexing with fluorene based H-acceptor polymer and carbazole based H-donor dyes. The H-donors composed of 3,6- and 2,7-functionalized electron-donating carbazole cores flanked by symmetrical thiophene linkers and cyanoacrylic acid as the end group (**83**–**86**) are shown in Figure 28. The H-acceptor polymer consists of side-chain carrying pyridyl pendants (**82**) [96]. DFT calculations have predicted that the carbazole units of H-donor are co-planar with the pi-spacer (conjugated thiophenes) and cyanoacrylic acids in the dyes. The supramolecular polymer networks were confirmed by FTIR. The complexes have a broad range of absorption in the visible range between 350 and 600 nm. As in the previous case, BHJ photovoltaic devices were fabricated using H-bonded polymers as donors and PC_61_BM as the acceptor. The highest efficiency was obtained for **82**/**84** (0.31%) which is attributed to broader absorption and increased photoinduced charge transfer as exhibited by the highest photoluminescence quenching. BHJ devices were also made by blending PC_61_BM with **82**, **84** and **85**, and the PCEs obtained were 0.02%, 0.15% and 0.11%, respectively. These values are significantly lower than those of H-bonded polymer networks of **82**/**84** (0.31%) and **82/85** (0.24%). Therefore, supramolecular H-interaction has proven to be a good method to enhance the PCE.

**Figure 29:**
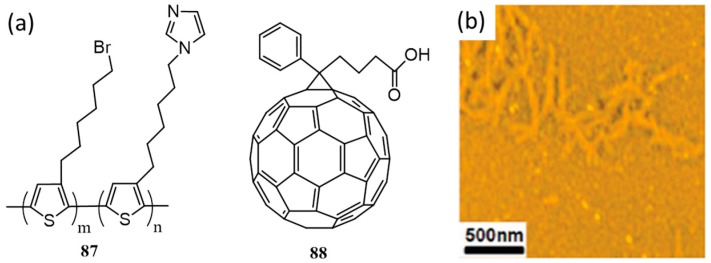
(**a**) Chemical structure of **87** and **88** synthesized by Chen et al. [97] (**b**) Tapping-mode AFM topographic image obtained from the surfaces of **87**/**88** (1:0.8 *w*/*w*) films prepared as-spun using CB/MeOH (49:1) as a solvent. Adapted with permission from Ref. [97] Copyright 2012 American Chemical Society.

An approach to control the molecular stacking through the intermolecular H-bonds induced by solvents was demonstrated by Chen et al. They have synthesized a regioregular poly{[3-(60-bromohexyl)thiophene]-*co*-[3-(6′-(1-imidazole)hexyl)thiophene]} (**87**; Figure 29a) for building supramolecular nanoscale domains in polymer solar cells [97]. Studies proved that the polymer forms stable H-bonding with methanol. The self-assembly properties of the polymer were also explored in the presence of **88** (Figure 29b). In chlorobenzene, no higher order aggregates were observed, indicating that the compounds were molecularly dissolved and no interaction occurs between them. However, when 2% *v*/*v* methanol was added, the absorption intensity enhances with a red-shift in the absorption maximum due to stronger pi-pi stacking. The AFM height images of blend films show fiber-like textures (Figure 29b), suggesting that aggregates are present in this composition. Polymer solar cells that were cast from CB/MeOH (49:1) gave an efficiency of 1.65%, which on annealing improved to 3.16%. The authors concluded that the hydrogen bonds involved in the system provide a delicate balance between self-assembly of **88** and crystallization of **87** resulting in the desired morphology and better efficiency.

**Figure 30:**
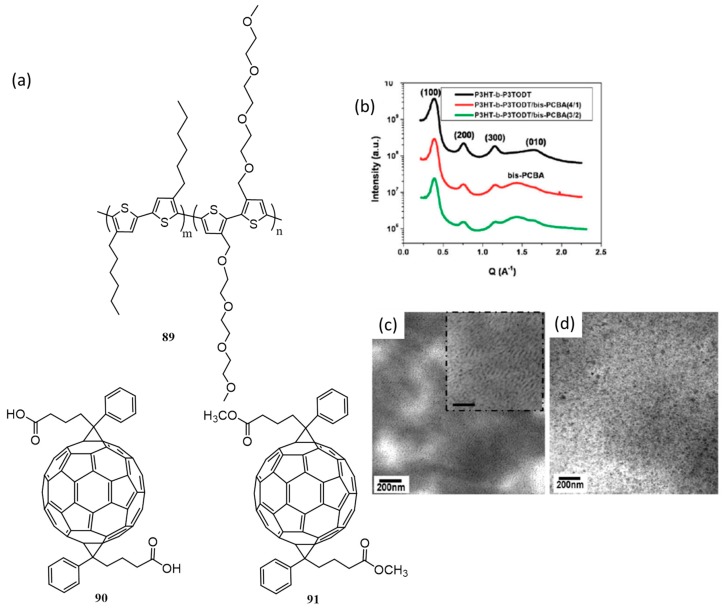
(**a**) Chemical structures of **89**–**91** synthesized by Watkins et al. [106]. (**b**) Overlay of GIXRD out-of-plane diffractograms of **89** and its blends with different ratio of **90**. TEM images of (**c**) **89**/**91** (3:2) and (**d**) **89**/**90** (3:2), after thermal annealing at 150 °C for 30 min. Abbreviated by authors as following: **89**:P3HT-*b*-P3TODT, **90**:bis-PCBA and **91**:bis-PCBM. Adapted with permission from Ref. [106] Copyright 2012 American Chemical Society.

Recently, block copolymers (BCP) emerge as a promising class of materials or templates for organic solar cells. BCPs consist of two or more covalently bound non-compatible homopolymers. The incompatibility of the blocks leads to morphological segregation in the film state but the large scale segregation will be prevented due to the presence of covalent bonds. This unique behavior of BCPs makes them an ideal candidate for solar cells, and many reviews are available on this topic [98,99,100,101,102,103,104]. Rod-coil BCPs of poly(3-hexylthiophene) and polystyrene, poly(2-vinyl pyridine), or polylactide have been studied extensively by many groups [105]. Watkins et al. synthesized block copolymer, (**89**) comprised of poly(3-hexylthiophene) (**18**) and poly[3-(2,5,8,11-tetraoxadodecane)thiophene] (P3TODT) and studied the cooperative self-assembly with functionalized fullerene derivatives (**90** and **91**) in BHJ solar cells (Figure 30a) [106]. The cooperative self-assembly happens through H-bond between the fullerene and one segment of the block copolymer. UV-visible absorption and GIXRD data revealed the pi-pi stacking of **18** inducing crystallinity to the copolymer. AFM images of the thin films of **89** reveal a periodic nanostructure with some degree of long-range order. To study the effect of **90** on the crystallinity and molecular packing of **89**, GIXRD experiments were conducted on blend films of varying weight ratios (Figure 30b). It was found that the blend has similar peaks to that of **89** films which were attributed to the ordered **18** microdomains. This suggests that **90** get incorporated in the amorphous domains of P3TODT. Comparison of TEM images was conducted on the blend samples of BCP with **90** and **91** (Figure 30c,d). A clear macrophase separation was seen in the **89**/**91** film, while the **89**/**90** system exhibits nanostructure morphology due to favorable H-bonding between the polymer and fullerene derivative. Field-effect transistor analysis revealed that the hole mobility of **89** was slightly lower than **18**. This could be due to the presence of amorphous P3TODT in the copolymer, which reduces the crystalline content compared to homo-**18**. BHJ cells were made from **89**/**90** (3:2) exhibited a higher PCE (2.04%), in comparison to those with the **89**/**91** (3:2) system (0.95%). For the **89**/**91** system, large domains of **91** (50–100 nm) were observed, which reduce the donor-acceptor interface available for charge separation and results in low device efficiency.

**Figure 31:**
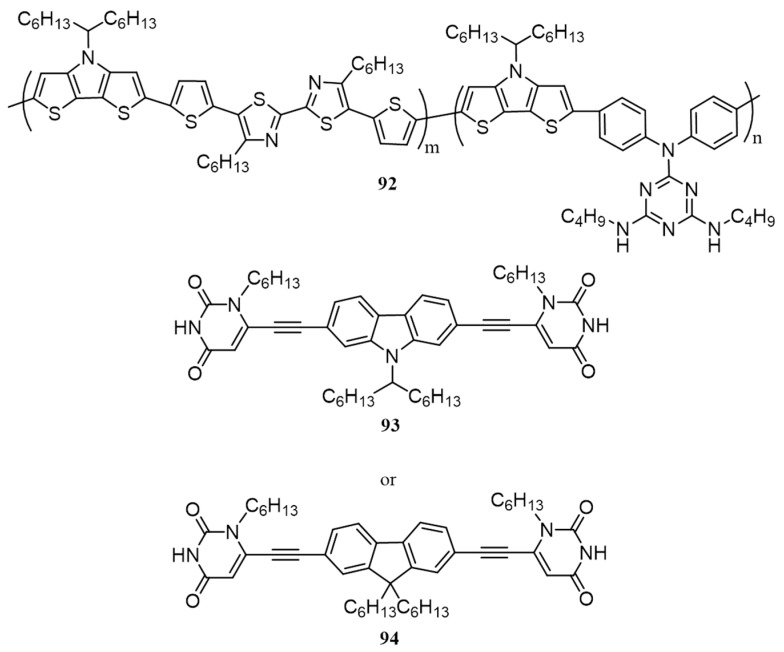
Chemical structure of the conjugated main-chain polymer (**92**) and complementary uracil-based conjugated cross-linkers (**93** and **94**), synthesized by Lin et al. [107].

Chu, Lin and co-workers have synthesized a low-bandgap polymer (**92**) consisting of bithiazole, dithieno[3,2-*b*:2′,3′-*d*]pyrroles, and pendent melamine derivatives [107]. Using the same method described by Lin et al. supramolecular polymers were made with complementary uracil-based conjugated cross-linkers containing carbazole (**93**) and fluorene (**94**). The structures of the polymer and the cross linkers are given in Figure 31. The supramolecular polymers (**92**/**93** and **92**/**94**) have enhanced properties in terms of light absorption, bandgap and crystallinity. The PCEs of fabricated devices, where the active layer was composed of supramolecular polymer networks as electron donors and PC_71_BM as an electron acceptor in the ratio 1:1 were found to be 0.97% and 0.68%, respectively. In contrast, a blend of polymer **92** with PC_71_BM yielded an efficiency of 0.52%. The PCE of **92**/**93** was further improved to 1.56% by changing the polymer/PC_71_BM ratio to 1:2 *w*/*w*.

**Figure 32:**
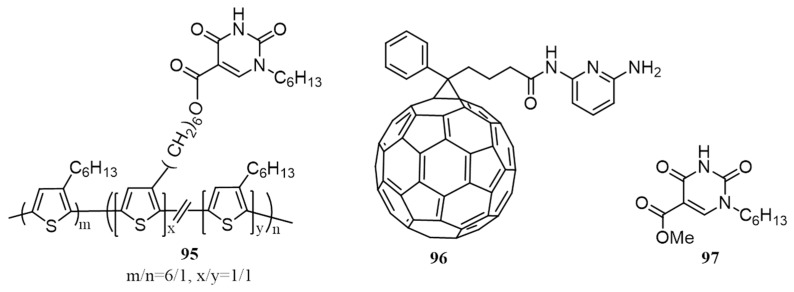
Chemical structures of the functionalized block copolymer (**95**), 2,6-diaminopyridine tethered fullerene derivative (**96**) and the model compound (**97**) synthesized by Qin et al. [108].

In another work utilizing the ‘three point’ complementary H-bonding, Qin et al. in 2013, synthesized 1-*n*-hexylisoorotic acid functionalized BCP (**95**) and 2,6-diaminopyridine tethered PC_61_BM analog (**96**; Figure 32) [108]. In order to study the complementary H-bonding between **95** and **96**, fluorescence titration experiments were carried out. Quenching of 26% in fluorescence intensity of **95** was observed, whereas, less than 7% decrease was seen for **18** under identical conditions. The Stern-Volmer constants obtained from quenching studies proved the presence of a strong binding between **95** and **96** in chlorobenzene at room temperature. The strength of the ‘three-point’ hydrogen bonding was further confirmed by ^1^H NMR titration experiments between **96** and **97** (**97** is a model compound) in CDCl_3_. The devices were fabricated with conventional structures by varying the weight ratios of polymer to the fullerene derivatives. It was observed that the composition of 10:8:0 as well 10:0:8 of **95**/**96**/PC_61_BM gave an efficiency of 2.65% and 2.36%, respectively under optimized conditions. It was obvious that the devices of 10:8:0 performed well due to the ‘three-point’ complementary interactions present in the donor/acceptor moieties.

**Figure 33:**
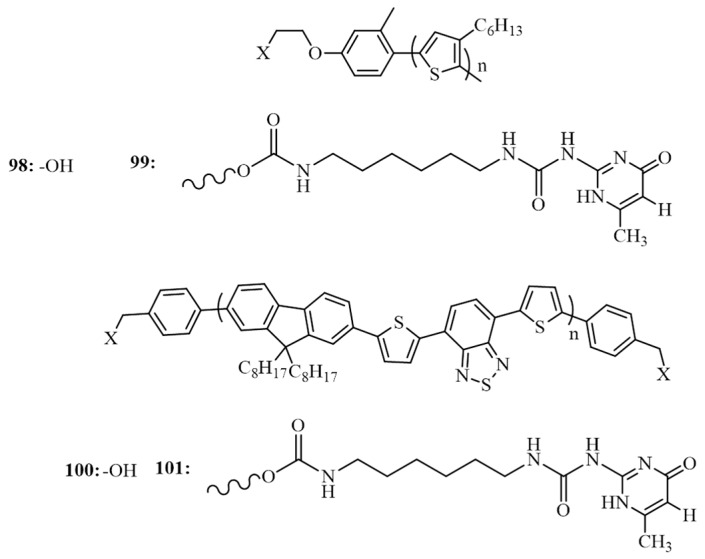
Chemical structures of **98**–**101** synthesized by Verduzco et al. [115].

Verduzco et al. have extensively studied the use of BCPs for photovoltaic applications [109,110,111,112,113,114]. Very recently, they reported the photovoltaic properties of a supramolecular BCP consisting of donor, poly (3-hexylthiophene) and acceptor, poly(2,7-(9′,9′-dioctyl-fluorene)-*alt*-5,5-(4′,7′-di-2-thienyl-2′,1′,3′-benzothiadiazole)) conjugated polymers connected through self-associating ureido-pyrimidinone hydrogen bonding units (Figure 33) [115]. Mixing of **98** and **100** containing hydroxyl groups form non-associating conjugated polymer blends while mixing of **99** and **101** containing ureido-pyrimidinone end group resulted in supramolecular BCP. BHJ cells were prepared using conventional device architecture and compared the performance of supramolecular BCP with that of the non-associating conjugated polymer blends under two different annealing conditions: 100 and 155 °C. The non-associative blends exhibited a decrease in PCE at higher annealing temperature (from 0.62% to 0.43%) while the supramolecular BCP exhibited an enhancement in performance (from 0.45% at 100 °C to 0.77% at 155 °C). Morphological analysis proved that the supramolecular association increased the miscibility and long-range ordering of P3HT crystallites at elevated temperatures which resulted in performance superior to non-associating conjugated polymer blends. The enhancement was also attributed to the inhibition of large scale macroscopic phase separation in supramolecular BCP.

**Figure 34:**
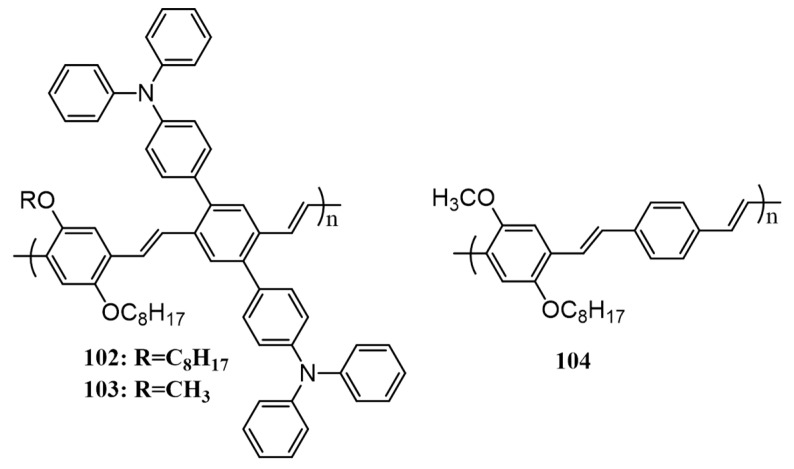
Chemical structure of the PPV derivatives (**102**–**104**) synthesized by Tan et al. [116].

Poly(*p*-phenylenevinylene) (PPV) is an attractive class of conjugated polymer for advanced device applications due to their interesting optical and electronic properties. Reports suggest that PPVs are good candidates for BHJ solar cells. Tan and co-workers in 2008 synthesized PPV derivatives consisting of triphenylamine moieties capable of 3D pi-pi stacking as side chains (**102** and **103**; Figure 34) [116]. For comparison, PPV without triphenylamine (**104**) was also synthesized. The BHJ solar cells in combination with fullerene (PC_61_BM) have been fabricated and characterized. The PCE of **103**/PC_61_BM (0.45%) was higher in comparison with that of **102**/PC_61_BM (0.27%), while **104** gave an efficiency of 0.09%. The results indicated that improved efficiency for **102** and **103** was due to the extended conjugated side chains of triphenylamine moieties by 3D pi-pi stacking, which was also reflected in the improvement in the hole-transporting properties of these polymers.

**Figure 35:**
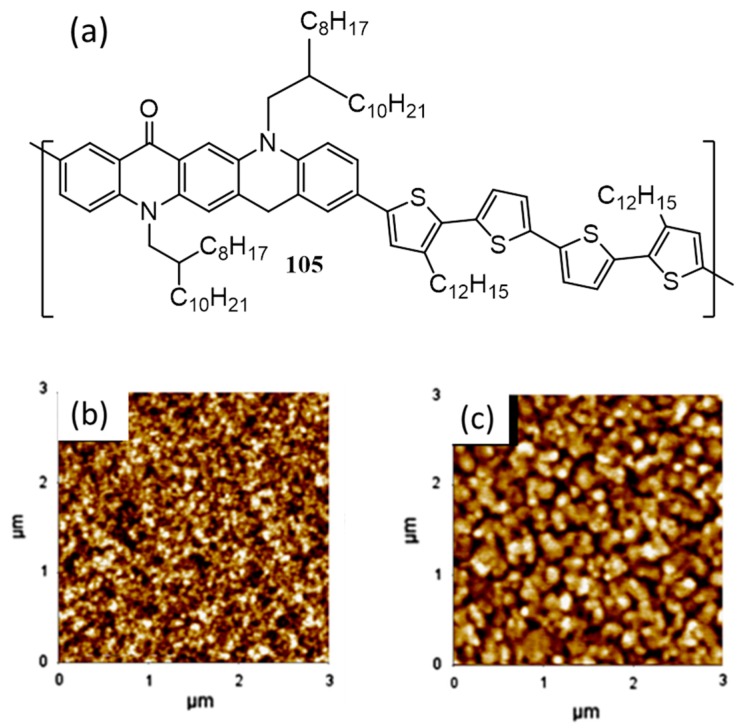
(**a**) Chemical structure of the polymer (**105**) synthesized by Moon et al. [118]. Topographic AFM images of (**b**) **105**/PC_71_BM 1:1 (3 × 3 μm^2^) and (**c**) **105**/PC_71_BM 1:2 (3 × 3 μm^2^). Adapted with permission from Ref. [118] 2013 Elsevier.

Quinacridone, commonly known as red–violet pigment is used for making organic thin film transistors with high mobility [117]. This planar molecule exhibit high crystallizability and excellent self-assembly properties. A novel poly[quinacridone–quaterthiophene] (**105**) containing quinacridone and quaterthiophene derivatives was synthesized by Moon et al. in 2008 (Figure 35a) [118]. It was expected that the compound would show efficient self-assembly and charge carrier mobility due to the presence of quinacridone core. Film state X-ray diffraction measurements were done to analyze the ordering structure of **105**. A d-spacing of 23.2 Å (λ = 2dsinθ), indicates that **105** formed the conventional edge-on pi-stacking on the substrate while the pi-pi stacking distance was found to be 3.5 Å. The photovoltaic device of the polymer with PC_71_BM (1:2) gave an efficiency of 2.3% and 2.0% in the presence and absence of poly[(9,9-bis(30-(*N*,*N*-dimethylamino)propyl)-2,7-fluorene)-*alt*-2,7-(9,9-dioctylfluorene)] (PFN), respectively. PFN is used to enhance the interfacial adhesion and electron transport properties between the active layer and cathode.

Interestingly, when the donor/acceptor weight ratio was changed to 1:1, the efficiency was dropped to 1.1% without PFN and 1.8% with PFN. In order to understand the difference in performance by varying the ratio of the active layer, hole mobility was measured using SCLC method. The hole mobility of **105**/PC_71_BM film of 1:1 ratio was estimated to be 4.70 × 10^−3^ cm^2^·V^−1^·s^−1^, while that of 1:2 ratio was 3.85 × 10^−3^ cm^2^·V^−1^·s^−1^. Since the electron mobility of PC_71_BM was 2 × 10^−3^ cm^2^·V^−1^·s^−1^, a better balance in the hole and electron transport happen only in 1:2 blended films. Apart from this, AFM images of the active layer with 1:1 ratio (Figure 35b) posed good micro-phase separation between polymer and PC_71_BM, whereas, the blend film showed self-assembled, large domains when the ratio was 1:2 (Figure 35c). The former morphology is good for a better charge carrier generation yielding higher efficiency.

**Figure 36:**
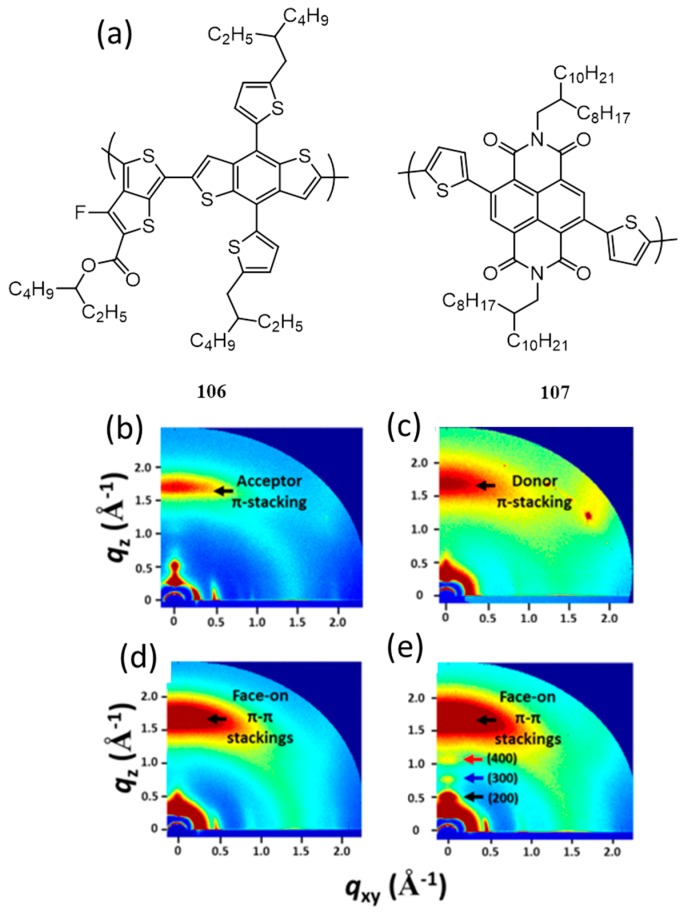
(**a**) Chemical structure of polymers (**106** and **107**) synthesized by Kim et al. [121]. 2D-GIXRD images of (**b**) **107**; (**c**) **106**, **106**/**107** blend (1.3:1) films (**d**) without DIO; and (**e**) with DIO. Adapted with permission from Ref. [121] Copyright 2014 American Chemical Society.

Kim et al. have reported that a low-bandgap polymer, poly[4,8-bis(5-(2-ethylhexyl)thiophen-2-yl)benzo[1,2-*b*:4,5-*b*′]dithiophene-*alt*-3-fluoro thieno [3,4-*b*] thiophene-2-carboxylate] (**106**) as the electron donor[119] and poly[[*N*,*N*′-bis(2-octyl dodecyl)- napthalene-1,4,5,8-bis(dicarboximide)-2,6-diyl]-*alt*-5,5′-(2,2′-bithiophene)] (**107**) as the acceptor[120] for BHJ solar cells [121]. The structures of the polymers are shown in Figure 36. Both donor and acceptor have large pi-plane to facilitate better stacking. From contact angle experiments, it was observed that the polymers have very similar surface tensions of 26.1 and 25.5 mN·m^−1^, respectively. Therefore, it was assumed that the polymer blends have the potential for forming well-intermixed morphology with suppressed phase separation as it possesses extremely low interfacial tension of 0.25 mN·m^−1^ between the domains of **106** and **107**. In order to understand the real potential, photovoltaic cells were fabricated with blends of donor/acceptor polymers (1.3:1). Cells with inverted architecture gave an efficiency of 4.60% with DIO (1.25 vol %), and 3.41% without DIO. For gaining a deeper understanding of the effect of DIO, charge-carrier mobilities of the blends were measured. A ten-fold increase in the electron mobility was observed for devices with DIO than that of devices without DIO. As a result, the ratio between the hole and electron mobilities in the former device were more balanced, which reduces the charge recombination thus yielding better photovoltaic performance. The direct evidence for the effect of DIO on the morphology of the polymer solar cells was obtained from 2D-GIXRD scattering analysis as shown in Figure 36b–e. The film of **107** showed strong (100), (200), and (300) peaks with pronounced reflections in both in-plane and out-of-plane directions, whereas, **106** showed only first order reflections. Upon blending of **106**/**107** in the absence of DIO, the disappearance of higher order peaks of **107** was observed. However, a remarkable recovery of the crystalline features of **107** along with a prominent pi-pi stacking peak with face-on geometry was observed in the presence of DIO. Highly ordered face-on stacks of the polymers treated with DIO led to the better charge transport from the active layer to the corresponding electrodes, eventually resulted in higher PCE in comparison to blends without DIO.

**Figure 37:**
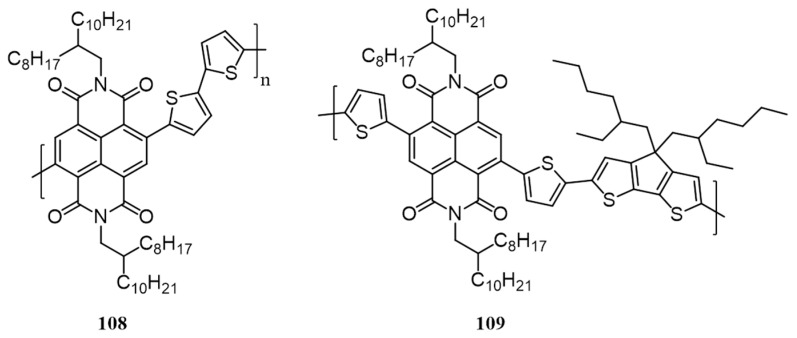
Chemical structure of copolymer acceptors (**108** and **109**) synthesized by Neher et al. [121].

As in the case of PDIs, naphthalenediimide (NDI) based systems also have a strong tendency to aggregate in specific organic solvents. BHJ active layers comprised of naphthalene derivatives often contain large aggregates resulting in poor efficiency. Neher et al. were able to successfully suppress this preaggregate formation at the early stages of film formation by using highly polarizable aromatic solvents such as *p*-xylene, chloronaphthalene and tetralin [121]. Prior to fabrication of solar cell the optical properties of the polymers were studied in different solvents (toluene, *p*-xylene, chlorobenzene, chloroform, dichlorobenzene, trichlorobenzene, tetralin and chloronapthalene). A high degree of preaggregation was observed in toluene and *p*-xylene, while aggregation was weaker or even absent in tetralin and chloronapthalene. The authors fabricated BHJ solar cells consisting of poly(3-hexylthiophene) (**18**) as donor and NDI copolymers (**108**, **109**) as acceptors (Figure 37). In the case of **108**, highest PCE (1.4%) was obtained for a mixture of *p*-xylene and chloronaphthalene (1:1), while **109** gave the highest efficiency (1.1%) when fabricated from tetralin.

## 4. Conclusions

Use of various supramolecular interactions is a proven way of making functional nanostructures with desired morphological features. In recent years, scientists have been using these weak non-covalent interactions for making organic solar cell devices with good stability. In this review, we have briefly discussed all works where supramolecular interactions were used effectively for making photovoltaic devices. Table 1 summaries the various photovoltaic parameters of the organic small molecules and polymers discussed in this review along with the corresponding references. These reports clearly suggest that supramolecular interactions and self-assembly are an impressive tool in tuning and tailoring the nanoscale domains of the active layer of organic solar cells. Both hydrogen bonding and pi-pi stacking improve the film quality and electronic communication between the chromophores. However, excessive aggregation of the chromophores through these interactions results in large scale domains, leading to poor exciton dissociation. Moreover, due to the increase in the surface roughness of the film, the charge carrier collection efficiency at the electrode interface also decreases, yielding poor power conversion efficiencies. No universal methods have been developed to address this issue and the control of the size of the nanostructures still remains a concern. However, rational selection of solvents, solvent additives and post-fabrication temperature treatments was found to be useful in certain cases. Limiting pi-stacking through the use of covalently fixed twisted dimers and bulky side chains are effective in non-fullerene acceptors. Another major issue is the lack of control over the direction of the self-assembly in systems for OPV applications. Chromophore packing perpendicular to the substrate/electrode surface is absolutely necessary for good efficiency. In summary, although supramolecular materials based technologies are ready for industrial applications, the use of supramolecular interactions in organic solar cells for commercial use needs more time as it is still in its infancy stage.

## Figures and Tables

**Table 1:** Photovoltaic parameters of the BHJ solar cells discussed in this review.

SI No	Device Structure	*J*_SC_ (mA·cm^−2^)	*V*_OC_ (V)	FF	η (%)	Light Intensity (mW·cm^−2^)	Ref.
1.	ITO/PEDOT:PSS/**1**:PC_61_BM(1:2.8)/Al	0.32	0.82	0.39	0.10	88	[48]
2.	ITO/PEDOT:PSS/**6**:PC_61_BM(1:1)/ZnO/Al	5.10	0.96	0.49	2.40	100	[51]
3.	ITO/MoO*_x_*/**9**:PC_71_BM(1:1)/LiF/PEDOT:PSS/Au	12.6	0.83	0.44	4.57	-	[52]
4.	ITO/PEDOT:PSS/**12**:PC_61_BM(1:1)/Ca/Al	7.00	0.79	0.54	3.01	100	[53]
5.	ITO/PEDOT:PSS/**17**:PC_61_BM(1:1)/Ca/Al	7.74	0.83	0.46	2.98	100	[54]
6.	ITO/PEDOT:PSS/P3HT:PC_61_BM(1:4)/Al	-	-	-	1.77	100	[55]
7.	ITO/PEDOT:PSS/**22**:PC_61_BM(1:1)/Al	0.84	0.47	0.46	0.18	100	[56]
8.	ITO/PEDOT:PSS/**23**:PC_61_BM(1:1)/Ca/Al	5.43	0.81	0.41	1.8	100	[57]
9.	ITO/PEDOT:PSS/**24**:PC_61_BM(1:0.7)/LiF/Al	9.79	0.82	0.54	4.31	100	[58]
10.	ITO/PEDOT:PSS/**31**:PC_61_BM(1:2)/Al	1	0.6	0.38	0.26	100	[59]
11.	ITO/PEDOT:PSS/**32**:PC_61_BM(1:1)/ZnO/Al	3.90	0.64	0.47	1.15	100	[60]
12.	ITO/**35**:**36**(40:60)/Al	0.03	0.69	0.40	1.95	0.47	[63]
13.	ITO/PEDOT:PSS/**38**:PC_71_BM(1:3)/LiF/Al	9.32	0.57	0.37	2.05	100	[64]
14.	ITO/PEDOT:PSS/**43**:PC_71_BM(2:1)/Al	8.3	0.76	0.58	4.1	100	[65]
15.	ITO/PEDOT:PSS/**46**:PC_71_BM(3:2)/LiF/Al	7.26	0.67	0.49	2.4	100	[67]
16.	ITO/ZnO/**51**:**52**(1:1)/MoO_3_/Ag	6.56	1.02	0.54	3.67	100	[74]
17.	ITO/ZnO/**53**:PC_61_BM(1:1)/MoO*_x_*/Ag	3.25	0.62	0.37	0.75	100	[75]
18.	ITO/MoO*_x_*/**56**:PC_71_BM(1:2)/LiF/Al	10.17	0.75	0.58	4.39	100	[78]
19.	FTO/bl-TiO_2_/**57**:**58**(3:1)/52/PEDOT:PSS/Au	0.28	0.39	0.38	0.04	100	[81]
20.	ITO/PEDOT:PSS/**59**:PC_71_BM(40:60)/LiF/Al	1.79	0.62	0.35	0.48	80	[83]
21.	ITO/PEDOT:PSS/**61**:PC_71_BM(1:1)/LiF/Al	2.98	0.66	0.27	0.53	100	[84]
22.	ITO/PEDOT:PSS/**64**:PC_61_BM(1:1)/Al	2.86	0.71	0.45	0.91	100	[85]
23.	ITO/MoO_3_/**68**:PC_71_BM(45:55)/Ba/Ag	10.5	0.99	0.49	5.10	23	[89]
24.	ITO/MoO*_x_*/**72**:PC_70_BM(70:30)/Al	0.78	14.4	0.59	6.70	100	[90]
25.	ITO/PEDOT:PSS/**76**/**80**:PC_61_BM(1:1)/Ca/Al	3.17	0.47	0.34	0.50	100	[95]
26.	ITO/PEDOT:PSS/**82**/**84**:PC_61_BM(1:1)Ca/Al	1.90	0.55	0.29	0.31	100	[96]
27.	ITO/PEDOT:PSS/**87**:**88**(1:0.8)/LiF/Al	9.11	0.67	0.51	3.16	100	[97]
28.	ITO/PEDOT:PSS/**89**/**90**(3:2)/LiF/Al	6.32	0.58	0.55	2.04	100	[106]
29.	ITO/PEDOT:PSS/**92**/**93**:PC_71_BM(1:2)/Ca/Al	7.16	0.60	0.36	1.56	100	[107]
30.	ITO/MoO_3_/**95**:**96**(10:8)/Al	8.59	0.59	0.52	2.65	100	[108]
31.	ITO/PEDOT:PSS/**99**:**101**(1:1)/Al	3.18	0.96	0.25	0.77	100	[115]
32.	ITO/PEDOT:PSS/**103**:PC_61_BM(1:1)/LiF/Al	1.20	0.89	0.38	0.45	90	[116]
33.	ITO/PEDOT:PSS/**105**:PC_71_BM(1:2)/Al	7.90	0.59	0.49	2.3	100	[118]
34.	ITO/PEDOT:PSS/**106**:**107**(1.3:1)/LiF/Al	10.61	0.82	0.53	4.60	100	[120]
35.	ITO/PEDOT:PSS/**18**:**108**(1:0.75)/Sm/Al	3.77	0.56	0.65	1.4	100	[121]
